# Host protease activity classifies pneumonia etiology

**DOI:** 10.1073/pnas.2121778119

**Published:** 2022-06-13

**Authors:** Melodi Anahtar, Leslie W. Chan, Henry Ko, Aditya Rao, Ava P. Soleimany, Purvesh Khatri, Sangeeta N. Bhatia

**Affiliations:** ^a^Harvard-MIT Division of Health Sciences and Technology, Institute for Medical Engineering and Science, Massachusetts Institute of Technology, Cambridge, MA 02139;; ^b^Koch Institute for Integrative Cancer Research, Massachusetts Institute of Technology, Cambridge, MA 02139;; ^c^Wallace H. Coulter Department of Biomedical Engineering, Georgia Institute of Technology and Emory School of Medicine, Atlanta, GA 30332;; ^d^Center for Biomedical Informatics Research, Stanford University, Stanford, CA 94305;; ^e^Graduate Program in Biophysics, Harvard University, Boston, MA 02115;; ^f^Microsoft Research New England, Cambridge, MA 02142;; ^g^Institute for Immunity, Transplantation and Infection, School of Medicine, Stanford University, Stanford, CA 94305;; ^h^Howard Hughes Medical Institute, Chevy Chase, MD 20815;; ^i^Department of Electrical Engineering and Computer Science, Massachusetts Institute of Technology, Cambridge, MA 02139;; ^j^Department of Medicine, Brigham and Women’s Hospital and Harvard Medical School, Boston, MA 02115;; ^k^Broad Institute of Massachusetts Institute of Technology and Harvard, Cambridge, MA 02142;; ^l^Hansjörg Wyss Institute for Biologically Inspired Engineering at Harvard University, Boston, MA 02115

**Keywords:** pneumonia, diagnostics, bacterial infections, viral infections, nanoparticles

## Abstract

Community-acquired pneumonia (CAP) has been brought to the forefront of global health priorities due to the COVID-19 pandemic. However, classification of viral versus bacterial pneumonia etiology remains a significant clinical challenge. To this end, we have engineered a panel of activity-based nanosensors that detect the dysregulated activity of pulmonary host proteases implicated in the response to pneumonia-causing pathogens and produce a urinary readout of disease. The nanosensor targets were selected based on a human protease transcriptomic signature for pneumonia etiology generated from 33 unique publicly available study cohorts. Five mouse models of bacterial or viral CAP were developed to assess the ability of the nanosensors to produce etiology-specific urinary signatures. Machine learning algorithms were used to train diagnostic classifiers that could distinguish infected mice from healthy controls and differentiate those with bacterial versus viral pneumonia with high accuracy. This proof-of-concept diagnostic approach demonstrates a way to distinguish pneumonia etiology based solely on the host proteolytic response to infection.

## Significance

Community-acquired pneumonia (CAP) is the most common infectious cause of death worldwide. In this work, we present a panel of protease-responsive nanosensors that leverage aberrant host protease activity in pneumonia to generate a urinary readout of disease. Notably, the urine signatures of host responses can also be used to differentiate between bacterial and viral pneumonia. These nanosensors constitute a possible route to diagnosing pneumonia that is orthogonal to existing clinical tests, thus opening a direction of study for pneumonia diagnostics.

At the start of 2020, the world was introduced to coronavirus disease 2019 (COVID-19), the disease caused by the novel severe acute respiratory syndrome coronavirus (SARS-CoV-2). This new form of community-acquired pneumonia (CAP) put pneumonia at the forefront of both medical research and public discourse. However, even before COVID-19, CAP had long been responsible for significant morbidity and mortality worldwide, with millions of people affected globally and over 100,000 deaths per year in the United States alone (1). The COVID-19 pandemic has demonstrated how the design and scaled production of new diagnostics that determine pneumonia etiology can benefit both individual patient management and public health. Unfortunately, the etiology of CAP in cases where SARS-CoV-2 is not the culprit remains hard to determine, and is often never identified (2, 3). Furthermore, clinical symptoms and radiologic parameters are insufficiently specific to distinguish between common bacterial and viral causes (4). As a result, the standard of care for patients with suspected CAP is to initiate empiric antibiotics as soon as possible based on local antibiotic resistance patterns and patient characteristics (e.g., age, comorbidities) (5), a strategy that may exacerbate antibiotic resistance and not provide clinical relief. To accurately triage, treat, and track patients with CAP due to bacterial and viral causes, new noninvasive tools that can both rapidly diagnose acute pneumonia and identify etiology must be developed.

Imaging via chest X-ray or computerized tomography is currently considered the gold standard pneumonia diagnostic, but such tools are known to suffer from significant deficiencies in specificity. Other previously routine tests, such as blood and sputum cultures, have fallen out of favor unless patients exhibit severe CAP (6). This change in protocol is due to the slow speed of cultivating bacterial isolates and distinguishing host flora from pathogens, during which time either treatment is delayed or patients are treated empirically (7). Furthermore, these cultures give no indication of viral etiologies, and the sample collection procedure is difficult, especially for pediatric patients. Urine antigen tests (UATs) are another means of detecting bacteria-driven pneumonia. These tests are both fast and noninvasive, but current UATs can only detect *Streptococcus pneumoniae* (SP) and *Legionella*, and thus will fail to identify the many other bacterial or viral causes of CAP (8). To diagnose viral pneumonia, rapid antigen tests are routinely performed for influenza, and molecular tests such as PCR offer high-sensitivity tools to detect other known viral causes. However, the cost per PCR sample can be high when rapid turnaround is needed, a known microbial sequence is required to produce a result, and sample collection methods can be uncomfortable or invasive. Notably, because PCR is an amplification-based method, the sensitivity of this assay can also lead to false positives, which has caused debate regarding proper threshold setting and its diagnostic capacity in the context of endemic exposure (9). Alternatively, rather than testing for the presence of a specific pathogen, host biomarkers that are associated with infection, such as circulating levels of inflammatory molecules, can be used to detect disease. For CAP, C-reactive protein (CRP) and procalcitonin (PCT) have been proposed to differentiate between bacterial and viral infections but ultimately suffer from poor sensitivity and have been shown to be insufficient to diagnose CAP, let alone distinguish etiology (4, 10).

Host response-based diagnostics have been developed that leverage the differential expression of host gene sets to distinguish bacterial versus viral infections (11, 12). These signatures mainly consist of genes that encode inflammatory markers, transmembrane proteins, and binding proteins that are implicated in the host immune response to infection but can only be made clinically useful by measuring their relative abundance through methods such as protein tests and gene sequencing. A potential trove of biomarkers lies within the over 550 human proteases that respond to, cause, and manage disease (13, 14). Proteases have well-established roles in cancer, vascular disease, apoptosis, and inflammation. In addition, they are now recognized as potential therapeutic targets for infectious disease, due to their production by invading pathogens as well as their involvement in the immune responses of an infected host (15, 16). Proteases are also attractive as biomarker targets since their enzymatic activity enables amplified detection by means other than simply measuring their absolute concentration in blood (14, 17). To this end, we have previously developed a category of nanomaterials that can be administered to a host in order to read out disease-specific enzymatic reactions through the generation of amplified, noninvasive “synthetic biomarkers” (18–24). These activity-based nanosensors (ABNs) are amenable for intrapulmonary delivery (25, 26) and detect protease activity in vivo to create urinary signatures of active disease. ABNs contain mass-encoded peptide linkers that are designed to be cleaved by proteases dysregulated in specific disease states. Upon peptide cleavage by a target protease, the linked barcodes are released from the ABN, after which they are small enough to diffuse into systemic circulation for subsequent renal concentration and clearance. By leveraging catalytic protease activity and concentrating barcodes from a large circulating volume of blood to a smaller urinary volume output, a highly amplified urinary readout is generated for sensitive disease detection. Furthermore, by multiplexing ABNs, one can create disease-specific urinary “signatures.”

**Fig. 1.**
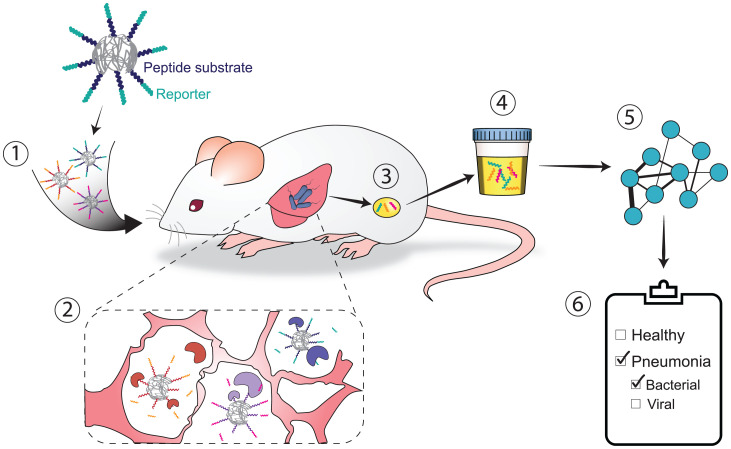
Schematic of how ABNs harness host-derived proteases to enable the diagnosis of pneumonia. 1) A multiplexed panel of ABNs with varying protease substrate linkers and corresponding mass-encoded reporters are administered to mice that have been infected with either bacterial or viral pneumonia. 2) Proteases are present in the lung cleave the ABNs at engineered substrate linkers, which release the reporters from the ABN scaffold (gray) into the circulation. 3) These reporters are filtered by the kidney and concentrated in the urine. 4) The reporters are then collected, and their concentrations are measured via mass spectrometry. 5) These concentrations are input into machine learning algorithms to train diagnostic classifiers. 6) This algorithm enables the diagnosis of pneumonia and, in the case of infection, specifies whether the etiology is bacterial or viral.

Here, using a previously developed computational framework (27), we have performed a multicohort analysis of blood transcriptome profiles from patients with a wide range of bacterial and viral respiratory infections to identify proteases predicted to be dysregulated based on infection etiology. This analysis yielded two unique host-derived gene signatures consisting solely of human proteases for bacterial and viral infection. We then demonstrated that a multiplexed panel of nanosensors designed to detect the activity of a subset of these enzymes could produce protease-driven urinary signatures to distinguish between bacterial and viral pneumonia in mouse models of CAP within 2 h of intrapulmonary sensor administration (Fig. 1). Using machine learning algorithms, we leveraged these urinary signatures to create diagnostic classifiers that can simultaneously detect CAP and distinguish etiology with high specificity and sensitivity. Thus, with this panel, we have created a proof of concept for a noninvasive urinary test for CAP that is driven by the biological host response to infection, rather than by detection of the pathogen itself.

## Results

### A 39-Gene Signature Distinguishes Bacterial and Viral Pneumonia.

**Fig. 2.**
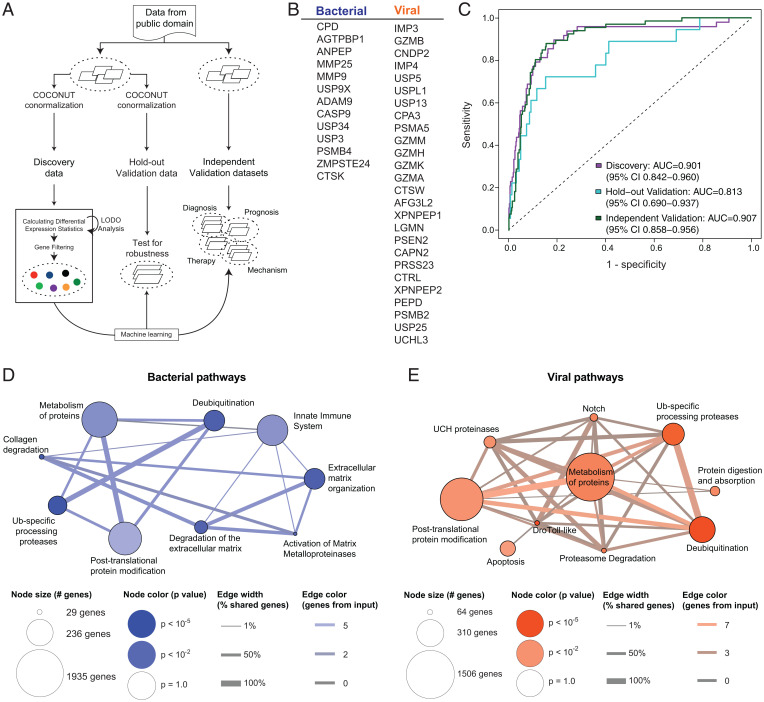
Generation of a bacterial versus viral infection protease signature using transcriptional metanalysis. (*A*) Publicly available transcriptional datasets from human patients with bacterial and viral respiratory infections were normalized using MANATEE, a computational framework for metanalysis of gene expression data. (*B*) MANATEE yielded a 39-gene signature of proteases that are differentially upregulated in bacterial versus viral infections. (*C*) A classifier was trained on human data from 16 published cohorts and validated on 17 independent published cohorts (*SI Appendix,* Tables S1 and S2). In total, 70% of samples (*n* = 495 nonhealthy samples) were used as discovery, and the other 30% were used in hold-out validation (*n* = 183 nonhealthy samples). ROC curves represent the distinguishing power of the classifier, where an AUC of 0.5 indicates the classifier performs as well as chance, and an AUC of 1 indicates perfect classification. (*D* and *E*) Biological pathways underlying the different gene sets were queried using a pathway analysis program (ConsensusPathDB). The pathways are represented by nodes, with the size indicating the number of total genes associated with that pathway and the color indicating the significance of the inputted gene set in terms of its association with the pathway. Signature genes that are shared between different pathways are depicted as edges, with the color indicating the number of shared input genes.

Given the plethora of human proteases, we hypothesized that the proteolytic host response to infection would be sufficiently distinct to distinguish between bacterial and viral pneumonia. To our knowledge, no disease-specific signatures consisting solely of host enzymes have been created to distinguish pneumonia etiology. To create such signatures for bacterial versus viral pneumonia, we curated publicly available transcriptomic datasets for respiratory infections from whole-blood and peripheral mononuclear blood cells (PBMCs), filtered these datasets for human peptidases (using the enzyme nomenclature term *ec:3.4.-.-*), and applied a computational multicohort framework designed to integrate gene expression data (multicohort analysis using aggregated gene expression [MANATEE]) (27) across 33 unique study cohorts (Fig. 2*A* and *SI Appendix*, Tables S1 and S2). By applying a set of differential expression statistics and machine learning algorithms, we identified a subset of 39 proteases that were consistently differentially expressed between bacterial and viral respiratory infections (Fig. 2*B*). With our previously described signature score model (11, 28), the expression of these protease genes was able to distinguish bacterial and viral respiratory infections in 16 discovery cohorts (area under the receiver operating characteristics curve [AUC] = 0.901, 95% CI: 0.842 to 0.960; Fig. 2*C*). Furthermore, in held-out, unseen samples from the 16 discovery cohorts, data from the 39 proteases achieved an AUC of 0.813 (Fig. 2*C*). Finally, in 17 completely independent cohorts, the expression pattern of these proteases maintained high accuracy in separating bacterial and viral respiratory infections (AUC = 0.907, Fig. 2*C*).

The proteases used in these signatures were selected purely based on differential gene expression and classification power. To validate their biological relevance to acute respiratory infections, we input the respective signatures into a molecular functions database (29, 30). Notably, the bacterial gene set was significantly associated with pathways that included innate immunity and extracellular matrix (ECM) organization and degradation (Fig. 2*D*). Neutrophils are an integral component of the early innate immune response and have been known to play a significant role in the clearing of bacterial pneumonia via mechanisms that include bacterial killing, antimicrobial peptide production, and recruitment of other innate immune cells (31). The ECM has also been shown to influence bacterial adhesion and colonization and is remodeled during tissue repair following inflammatory damage (32). Furthermore, the viral protease set was significantly enriched in several different pathways, including apoptosis, which is a classic defense mechanism against viral infections (33) (Fig. 2*E*). The associations between these newly derived in silico signatures and known biological pathways gave us confidence that our 39-protease gene signature (ProSet) would prove to include active, functional players in pneumonia and, thus, valuable biomarkers in vivo.

### Proteases Involved in Lung Infections Inform the Creation of a Pneumonia-Specific Panel of ABNs.

ABNs produce a functional output for disease and possess an inherent amplification factor, in that a single target enzyme can trigger the release of multiple reporters. Therefore, rather than rely on endogenous gene expression, we sought to create ABNs that leverage the catalytic activity of host proteases to generate a noninvasive and amplified urinary readout to distinguish bacterial versus viral pneumonia. The ProSet provided a foundational list of pneumonia-specific biomarkers that could be targeted by our ABNs. To create suitable ABNs, we sought to identify peptide substrates that were susceptible to cleavage by these enzymes for use as protease-cleavable linkers. Some enzymes in the ProSet had cleavage profiles that were unsuitable for targeting by ABNs (e.g., aminopeptidases), and others did not have suitable mouse homologs (e.g., GZMH). For the remaining viable protease candidates, we drew from prior literature to identify peptide substrates that had been previously optimized (see Table 1 for sequence source information). We then expanded the substrate pool by drawing upon our previous work, in which we identified peptide sequences that are efficiently cleaved in mouse models of diseases involving lung inflammation, such as lung cancer and bacterial pneumonia (21, 23, 25, 26), and nominated candidates based on literature review (references in Table 1). This process resulted in a panel of 20 substrates, which were individually conjugated to a 40-kDa polyethylene glycol (PEG) core to form 20 distinct ABNs (Table 1). The N terminus of the linker was synthesized with a mass-encoded glutamate-fibrinopeptide B (GluFib) reporter, a peptide that is stable in circulation and readily renally cleared, to enable detection in urine via mass spectrometry. However, the N terminus can also accommodate other reporter molecules, such as a fluorophore or short peptide domain, to suit different readout modalities (34, 35).

**Table 1.** 20-plex panel of ABNs for pneumonia

Name	Sequence (reporter, peptide sequence)	Sequence source
BV01	*^e(+2G)(+6V)ndneeGFFsAr^* ^-(ANP)-^ **^GGAIEFDSGC^** ^-(PEG8-40kDa)^	Published GZMB substrate (36)
BV02	*^eG(+6V)ndneeGF(+1F)s(+1A)r^* ^-(ANP)-^ **^GGHPGGPQC^** ^-(PEG8-40kDa)^	Commercially available CATK substrate
BV03	*^e(+3G)(+1V)ndneeGFFs(+4A)r^* ^-(ANP)-^ **^GGGVFRMLSVGC^** ^-(PEG8-40kDa)^	Derived GZMA substrate (37)
BV04	^e^ *^(+2G)Vndnee(+2G)FFs(+4A)r^* ^-(ANP)-^ **^GGGLFRSLSSGC^** ^-(PEG8-40kDa)^	Screened GZMA substrate (37)
BV05	^eGVndnee(+3G)(+1F)Fs(+4A)r-(ANP)-^ **^GGGLLYGKGGC^** ^-(PEG8-40kDa)^	Published CAPN2 substrate (38)
BV06	*^e(+2G)(+6V)ndnee(+3G)(+1F)(+1F)s(+1A)r^* ^-(ANP)-^ **^GGy-Tic-TNGC^** ^-(PEG8-40kDa)^	Derived LGMN substrate (39)
BV07	*^eG(+6V)ndnee(+3G)(+1F)Fs(+4A)r^* ^-(ANP^ *^)-^* **^GGfPRSGGGC^** ^-(PEG8-40kDa)^	(26)
BV08	*^e(+3G)(+1V)ndneeG(+10F)FsAr^* ^-(ANP)-^ **^GGGSGRSANAKGC^** ^-(PEG8-40kDa)^	(26)
BV09	*^e(+2G)Vndnee(+2G)F(+10F)sAr^* ^-(ANP)-^ **^GGGIQQRSLGGGC^** ^-(PEG8-40kDa)^	(21)
BV10	*^eGVndneeGF(+10F)s(+4A)r^* ^-(ANP)-^ **^GGIPSIQSRGLGC^** ^-(PEG8-40kDa)^	Influenza hemagglutinin mimic peptide
BV11	*^e(+2G)(+6V)ndneeG(+10F)(+1F)s(+1A)r^* ^-(ANP)-^ **^GGNLARALKQTIGC^** ^-(PEG8-40kDa)^	Screened MMP substrate (40)
BV12	*^eG(+6V)ndneeG(+10F)Fs(+4A)r^* ^-(ANP)-^ **^GGHMVQHLIQWHGC^** ^-(PEG8-40kDa)^	Screened MMP substrate (40)
BV13	*^e(+3G)(+1V)ndnee(+2G)(+10F)Fs(+4A)r^* ^-(ANP)-^ **^GGPRAAA-Homophe-TSPGC^** ^-(PEG8-40kDa)^	Screened ADAM9 substrate (41)
BV14	*^e(+2G)Vndnee(+3G)(+10F)(+1F)s(+4A)r^* ^-(ANP)-^ **^GGTGPPGYTGC^** ^-(PEG8-40kDa)^	Screened ADAMTS substrate (42)
BV15	*^eGVndneeG(+10F)(+10F)sAr-(^* ^ANP)-^ **^GGTGLPVYQGC^** ^-(PEG8-40kDa)^	Screened ADAMTS substrate (42)
BV16	*^e(+2G)(+6V)ndnee(+3G)(+10F)(+1F)s(+4A)r-(^* ^ANP)-^ **^GG-Nle(O-Bzl)-Met(O)2-Oic-Abu-C^** ^-(PEG8-40kDa)^	Published NE substrate (43)
BV17	*^eG(+6V)ndneeG(+10F)(+10F)sAr-^* ^(ANP)-^ **^GGAAFAGC^** ^-(PEG8-40kDa)^	Published NE substrate (23)
BV18	*^e(+3G)(+1V)ndnee(+2G)(+10F)(+10F)sAr-(^* ^ANP)-^ **^GGGGGPGC^** ^-(PEG8-40kDa)^	(21)
BV19	*^e(+2G)VndneeG(+10F)(+10F)s(+4A)r-^* ^(ANP)-^ **^GGPLGMRGGC^** ^-(PEG8-40kDa)^	(26)
BV20	*^eGVndnee(+2G)(+10F)(+10F)s(+4A)r-ANP^* ^-^ **^GGP-(Cha)-G-Cys(Me)-HAGC^** ^-(PEG8-40kDa)^	(26)

Each nanoparticle consists of a mass-encoded GluFib reporter (in italics, d-amino acids represented by lowercase letters) attached to a photolabile linker (3-amino-3-(2-nitro-phenyl)propionic acid, ANP). The adjacent peptide substrate (in bold) is conjugated to an inert 40-kDa 8-arm PEG scaffold. All substrate sequences were derived from published studies.

### The Pneumonia ABN Panel Generates Etiology-Specific Urinary Signatures.

**Fig. 3.**
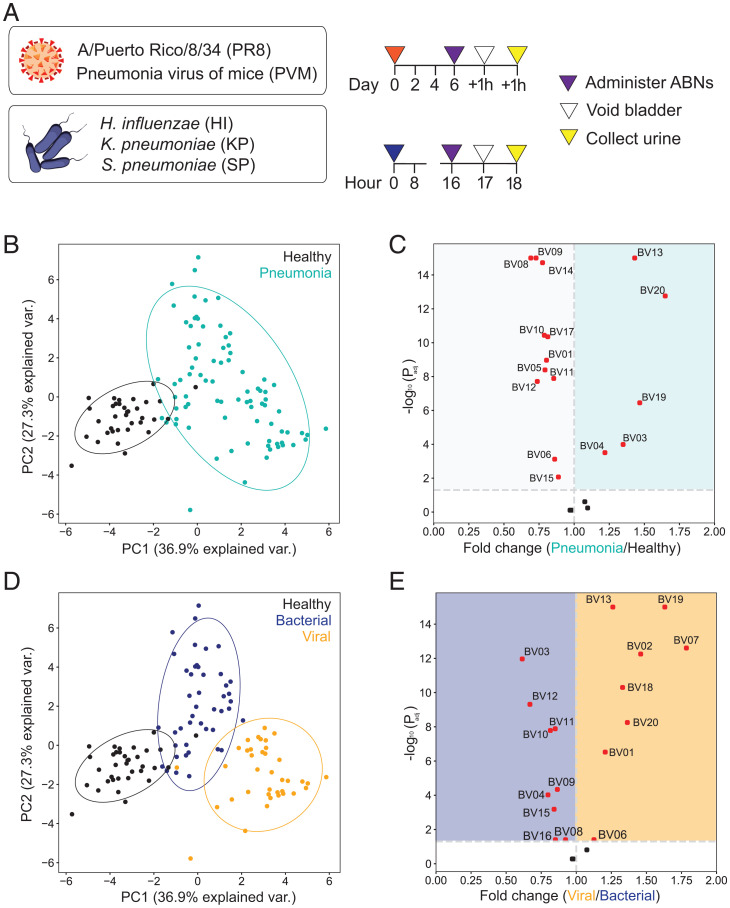
Nanosensors distinguish pneumonia and its etiology in mice. (*A*) ABNs were administered into five mouse models of pneumonia. Urine from each mouse was collected 2 h after administration to characterize in vivo ABN activity. (*B* and *D*) Unsupervised PCA of normalized urinary reporter concentrations in pneumonia (*n* = 83 mice) and healthy controls (*n* = 35 mice). Data from pneumonia mice are labeled according to either infection (*B*) or etiology (*D*) (bacterial pneumonia, *n* = 45; viral pneumonia, *n* = 38). (*C* and *E*) The relative fold change between disease states was calculated using mean-scaled reporter concentrations. Dotted vertical line represents no fold change between disease states. Each point represents one reporter, with significantly differential reporters in red (above the dotted horizontal line at Padj = 0.05). Significance was calculated using two-tailed *t* test with Holm-Sidak correction.

We next sought to determine whether this ABN panel could distinguish bacterial from viral pneumonia in vivo (Fig. 3*A*). We established five distinct mouse pneumonia models that represent common causes of CAP in humans by infecting immunocompetent BALB/c mice (see *Materials and Methods* for infection protocol) with either bacteria (SP, *Klebsiella pneumoniae* [KP], *Haemophilus influenzae* [HI]) or viruses (influenza A/PR/8/34 [H1N1] [PR8]; pneumonia virus of mice [PVM]). We optimized the dose of each pathogen to cause similar timelines of disease within each etiology based on lung bacterial and viral loads, as well as physical signs of illness, including weight loss and ruffled fur (*SI Appendix*, Fig. S1). To characterize the performance of our panel in vivo, we then delivered the 20 ABNs directly into the lungs of mice with bacterial pneumonia, viral pneumonia, or healthy controls and collected urine 2 h after administration.

After normalizing the urinary reporter levels relative to the administered stock and across each other (see *Materials and Methods* for details), we compared the concentrations to assess whether the panel of ABNs was differentially cleaved among disease states. Principal-component analysis (PCA) revealed a divergence between the infected mice and the corresponding healthy controls, consistent with the hypothesis that ABNs are differentially cleaved between these groups (Fig. 3*B*). Furthermore, the tight clusters indicated that a consistent set of protease cleavage events within each group gave rise to the pattern of reporters detected in urine. To define these signatures, we examined the relative differences in reporter concentrations between the healthy and infected mice and found that 17 of the 20 ABNs were significantly differentially cleaved in one state versus the other (Fig. 3*C*). This result demonstrated that the host protease response could be queried to generate a functional readout of active pneumonia. Furthermore, we noted that although the infected lung is presumably more protease-rich than healthy tissue, many reporters are significantly enriched in healthy controls relative to the pneumonia-infected mice, which highlights differential protease activity between the two states.

Relabeling the infected mice based on etiology revealed further separation between bacterial and viral pneumonia (Fig. 3*D*). Notably, of the five reporters enriched in the urine of infected mice with pneumonia compared to controls, three (BV13, BV19, BV20) were significantly enriched in the viral mice, while the other two (BV03 and BV04) were enriched in the bacterial mice (Fig. 3*E*). Reporters from additional ABNs (e.g., BV01, BV10, BV12) also emerged as differentially enriched in bacterial and viral pneumonia, even though they were enriched in the healthy controls relative to the pool of all infected mice. This observation that different sets of reporters were enriched based on context demonstrated that the multiplexed ABN panel yielded discrete reporter sets to reflect various disease states. Overall, these results revealed distinct differences in the urinary reporter concentrations, and thus in vivo protease activity, between mice with bacterial and viral pneumonia.

### ABNs Are Cleaved by a Wide Range of Protease Classes.

**Fig. 4.**
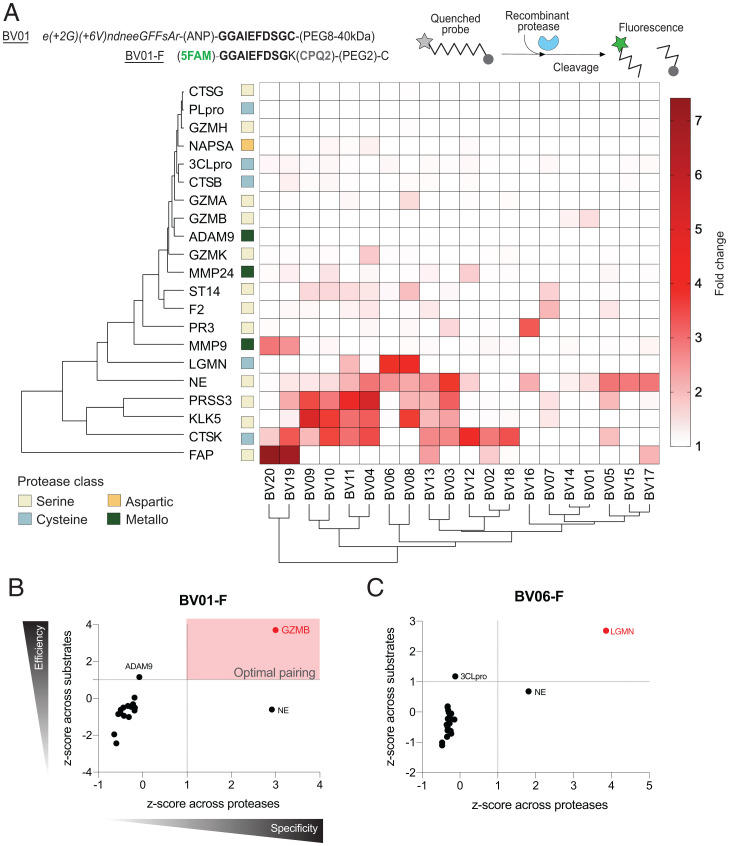
In vitro screening of fluorescent substrates reveals possible nanosensor targets. (*A*) The peptide sequence of each ABN was incorporated into a quenched fluorescent substrate. These fluorogenic probes were then incubated with recombinant proteases to evaluate cleavage profiles. Hierarchical clustering was performed based on the fold change in fluorescence after 10 min (average of two replicates). A fold change of 1 indicates no cleavage (white squares in the heat map); increased cleavage corresponds to higher color intensity. (*B* and *C*) Standardization was performed to assess protease-substrate pairings from the in vitro screening data. *Z*-scores of the average fold change values for each pairing across the proteases (*x* axis) and substrates (*y* axis) were compared using sSvE plots to characterize protease-substrate pairs with highly specific and efficient cleavage.

After the discovery of these in vivo cleavage signatures, we next sought to predict which proteases were driving the differential reporter enrichment based on the specificity of each substrate for its target protease. To screen the cleavage of each linker across a nonexhaustive range of proteases, we first reformulated each ABN into a fluorescent probe format by flanking the peptide substrate sequence with a fluorophore-quencher pair (Fig. 4*A*; sequences listed in *SI Appendix*, Table S3). We then incubated each probe with commercially available recombinant proteases that were either based on the ProSet or predicted to be present in the lungs (protease and buffer conditions in *SI Appendix*, Table S4). All probes were cleaved by at least one protease within 10 min, and several proteases known to be relatively promiscuous, such as neutrophil elastase (NE), exhibited particularly robust and broad substrate cleavage (Fig. 4*A*). We performed hierarchical clustering to determine which proteases produced orthogonal cleavage patterns and saw similar cleavage profiles across cathepsin K (CTSK), kallikrein 5 (KLK5), and serine protease 3 (PRSS3). However, we observed no obvious clustering based on protease class. Instead, most proteases gave rise to relatively distinct cleavage patterns across the panel of substrates, consistent with our rational approach to substrate selection.

Notably, while the peptide substrates were selected based on published susceptibility to cleavage by our proteases of interest, peptide sequences are often cleavable by multiple enzymes to varying degrees; thus, there will inevitably be substrate cleavage by additional proteases in vivo, especially in the protease-rich microenvironment of the infected lung. To parse through this confounding cleavage and more directly correlate our in vivo results with specific protease activity, we normalized the heat map data to identify putative specific protease-substrate pairs from our screen. We reasoned that standardizing across substrates would allow us to compare how quickly an individual protease cleaved one probe relative to others, thus providing a yardstick for cleavage efficiency. Conversely, normalizing across proteases reflected specificity by comparing the cleavage rates of one probe by a wide panel of proteases. To correlate these metrics, we combined them to create a specificity vs. efficiency (SvE) plot, which enabled the identification of optimal protease-substrate pairs that had both robust and specific cleavage (Fig. 4*B*). Based on their cleavage specificity and efficiency, several optimal protease-substrate pairs emerged from the screen. Groupings such as granzyme B (GZMB) with BV01-F (Fig. 4*B*) and legumain (LGMN) with BV06-F (Fig. 4*C*) confirmed that some of the rationally designed probes were being well cleaved by their intended targets. Other probes yielded no optimal protease hits (all plots available in *SI Appendix*, Fig. S2), potentially indicating that the ideal protease was not included in the screen or that the optimal cleavage kinetics for that probe were not achieved during a 10-min assay. Overall, while this analysis indicated that each ABN is vulnerable to protease cleavage, the presence of any given reporter in the urine may not directly correspond to the in vivo activity of a single protease.

### BV01 Signals Differences in the Host Immune Response to Bacterial and Viral Pneumonia.

After we observed differential cleavage of the ABNs in vivo and characterized cleavage susceptibilities in vitro, we sought to validate that our urinary reporter signatures were reflective of divergent host responses between bacterial and viral pneumonia. To do this, we focused on BV01, which was designed and validated to be cleaved by GZMB. GZMB is a serine protease that is produced by natural killer (NK) cells and cytotoxic T lymphocytes and has been implicated in the antiviral response (44). NK cells in particular are known to play an important role in antiviral immunity (45). However, there is evidence that this population can exert a detrimental effect on the lungs of immunocompromised mice in the context of SP via inflammatory cytokine production (46), suggesting that NK cells may also participate in the immune response to bacterial pneumonia.

**Fig. 5.**
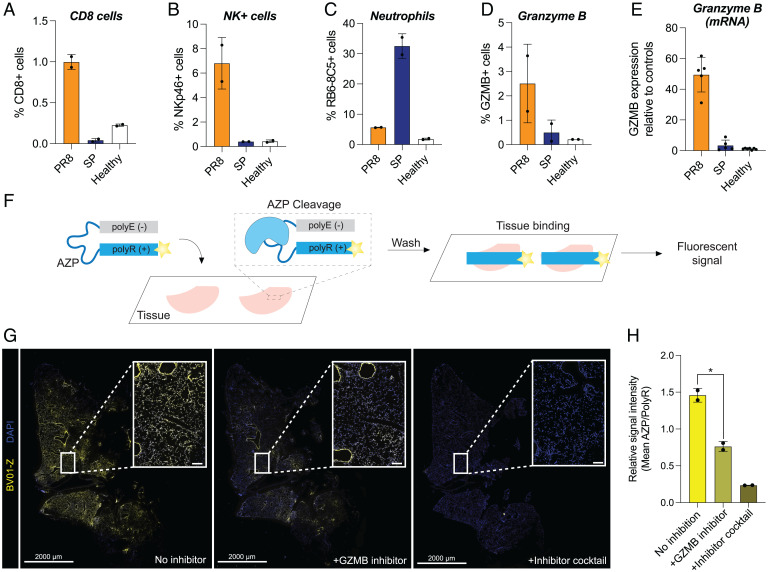
GZMB is elevated in viral pneumonia and contributes to nanosensor signal. (*A*–*D*) Percentage of detected cells positive for various cell markers via immunofluorescent staining performed on fresh-frozen sections from mice infected with either PR8 or SP (*n* = 2 consecutive sections per group, mean ± SD). Counts were obtained using QuPath, and stain-positive cells were identified via manually set thresholds. Counts for positive/total cells per section in each panel are (*A*) CD8 (PR8: 884/83153, 747/80149; SP: 49/86308, 34/127753; healthy: 252/117485, 289/122812); (*B*) NKp46 (PR8: 4256/80149, 6895/83153; SP: 345/86308, 505/127753; healthy: 593/117485, 421/122812); (*C*) RB6-8C5 (PR8: 5021/90127, 5549/97327; SP: 38217/128902, 51305/145035; healthy: 2641/130332, 2032/128009); and (*D*) GZMB (PR8: 1127/82380, 3432/94145; SP: 168/119890, 767/89157; healthy: 335/156523, 291/135411). (*E*) Relative expression of GZMB in lung tissue from healthy control mice and those with PR8 or SP via qRT-PCR. (*F*) The original BV01 substrate was incorporated into the AZP BV01-Z, consisting of the substrate sequence linking a fluorescently labeled polyR domain and a polyE domain. The AZP is applied to fresh-frozen tissue and is cleaved by active tissue-resident enzymes, after which the liberated polyR domain electrostatically binds to the tissue. (*G*) The GZMB responsive AZP BV01-Z (yellow) was applied to PR8-infected tissue with and without a GZMB-specific inhibitor or a broad-spectrum mixture of protease inhibitors. Sections were costained with a free polyR binding control (not shown) and counterstained with DAPI (blue). Staining shows one section of the slide, with white squares marking the location of the inset images. Scale bars for full sections: 2,000 μm; scale bars for zoomed regions: 100 μm. (*H*) Quantification of relative BV01-Z intensity in sections stained with or without inhibitors (*n* = 2 consecutive sections, mean ± SD; one-way ANOVA with multiple comparisons and Brown-Forsythe and Welch’s correction, *P = 0.0278).

To determine whether our differential BV01 signal was driven by the immune response to viral infection, we focused on two main causes of viral and bacterial pneumonia: PR8 and SP, respectively. We hypothesized that the virally associated BV01 signal we observed in vivo was due to increased recruitment of cells that produce GZMB in the lungs during viral infection. We assessed the presence of NK cells, which are known to be actively recruited to the lungs following influenza infection in mice (47), and cytotoxic T cells, via immunofluorescent staining for NKp46 (for NK cells) and CD8 (for T cells). We observed very small populations of antigen-expressing cells in the SP-infected tissue (Fig. 5 *A* and *B* and *SI Appendix*, Fig. S3). However, PR8-infected lungs had a higher number of positively stained cells, which suggests NKp46-expressing and CD8-expressing cells are recruited to the PR8-infected lungs. As a positive control, we also stained sections with a Ly6G-binding antibody (RB6-8C5) to mark neutrophil lineage cells. In contrast to the NKp46 and CD8 staining patterns, we observed robust staining of RB6-8C5+ cells in sections that were infected with SP versus PR8 (Fig. 5*C* and *SI Appendix*, Fig. S4). We also stained for GZMB protein in the same sections and saw increased expression in PR8-infected lungs relative to SP-infected samples (Fig. 5*D* and *SI Appendix*, Fig. S4), consistent with elevated GZMB levels by qRT-PCR in PR8 tissue samples (Fig. 5*E*). Given that there are circumstances in which NK cells can express CD8, at this resolution, we cannot exclude the possibility that the CD8 antibody may mark NK cells as well (48, 49). Nonetheless, we observed that NKp46+, CD8+, and GZMB+ cells are significantly more prevalent in virus-infected lungs compared to bacterial infection, which reinforces our model that urinary signatures generated by host cell responses vary based on infection etiology.

Collectively, our data led us to nominate GZMB as a viral target via transcriptomics; indicated that BV01 is efficiently and specifically cleaved by GZMB in vitro; suggested, based on immunostaining, that the cells producing GZMB are enriched in lungs infected with PR8 (viral pneumonia) compared to SP (bacterial pneumonia); and validated that GZMB itself is upregulated in a model of influenza based on immunohistochemistry and mRNA expression. We next sought to determine whether GZMB activity was the source of the viral-associated BV01 signal that was observed. To do this, we constructed an activatable zymography probe (AZP) based on the BV01 substrate sequence (BV01-Z) to assay for in situ GZMB cleavage in fresh-frozen lung tissue samples from our mouse models of infection (50–52). AZPs are composed of an anionic poly-glutamic acid (polyE) domain that is connected to a fluorophore-labeled cationic poly-arginine (polyR) domain via a peptide substrate (Fig. 5*F*). The AZP is applied topically to tissue similarly to the typical workflow for immunofluorescence, except when the peptide substrate connecting the polyE and polyR domains of the AZP (in this case, the BV01 substrate; sequence in *SI Appendix*, Fig. S5) is cleaved by a protease, the AZP is activated as the polyE and polyR domains separate (Fig. 5*F*). The liberated, fluorophore-labeled, cationic polyR domains can then bind locally to the tissue where the AZP was activated, thus labeling the site of protease activity in situ.

To confirm that BV01-Z was cleaved by GZMB and that proteolytic activation was necessary for tissue binding, we incubated the AZP with recombinant GZMB, allowing protease-driven activation to take place in vitro. We then applied either the precleaved mixture or intact AZP probe onto fresh-frozen sections of healthy mouse lung at a temperature that enabled tissue binding while preventing activity of endogenous enzymes. A fluorescent signal was visible in the sections incubated with the precleaved AZP, but not with the intact probe, supporting the hypothesis that that the AZP could be cleaved by recombinant GZMB and that a positive signal is dependent on protease activity (*SI Appendix*, Fig. S5).

Having validated that the GZMB-cleaved BV01-Z could bind to healthy lung tissue sections, we applied the intact AZP to fresh-frozen lung sections from PR8-infected mice to assay whether the reporter would be activated in situ and where it would bind. In these conditions, we observed strong BV01-Z labeling throughout the tissue (Fig. 5*G*). To determine whether this AZP activation signal could be attributed to GZMB activity, we then added a GZMB-specific inhibitor (Z-AAD-CH2Cl; in vitro validation of inhibition in *SI Appendix*, Fig. S5) to the tissue incubation step and observed a significant decrease in binding of the activated polyR domain (Fig. 5 *G* and *H*; *P = 0.0278), confirming that in situ GZMB activity contributes to BV01-Z activation. However, at least at this level of GZMB inhibition (*SI Appendix*, Fig. S5), some AZP signal remained. Notably, peptide substrates can exhibit broad susceptibility to proteolytic cleavage; this includes BV01, which we observed to be cleaved to a lesser extent by both NE and ADAM9 in vitro (Fig. 4*B*). Moreover, there are many proteases present in vivo, but only a small subset was tested in our in vitro cleavage screen.

Thus, we hypothesized that off-target BV01 cleavage by other tissue-resident proteases was responsible for the observed residual BV01 AZP signal in PR8-infected sections. Consistent with this prediction, when we incubated with a broad-spectrum mixture of serine, cysteine, metallo, amino, and aspartic peptidase inhibitors, the BV01-Z staining was further diminished relative to GZMB-specific inhibition, albeit not at a level that reached statistical significance (Fig. 5*H*; n.s., *P* = 0.0937). Having confirmed that GZMB did activate BV01-Z in situ, we then applied BV01-Z to both a whole-lung section of PR8 and a lobar section of healthy lung and observed elevated AZP signal in the infected tissue compared to healthy (*SI Appendix*, Fig. S6). Taken together, these results demonstrate that there is a greater influx of NK and CD8 T cells in viral pneumonia compared to bacterial pneumonia. These cells produce the viral pneumonia marker GZMB, which can cleave the BV01 peptide sequence in tissue sections from mice with viral pneumonia and, by proxy, in vivo to produce a reporter signal that is detectable in urine.

### The ABN Panel Can Classify Pneumonia and Determine Etiology.

**Fig. 6.**
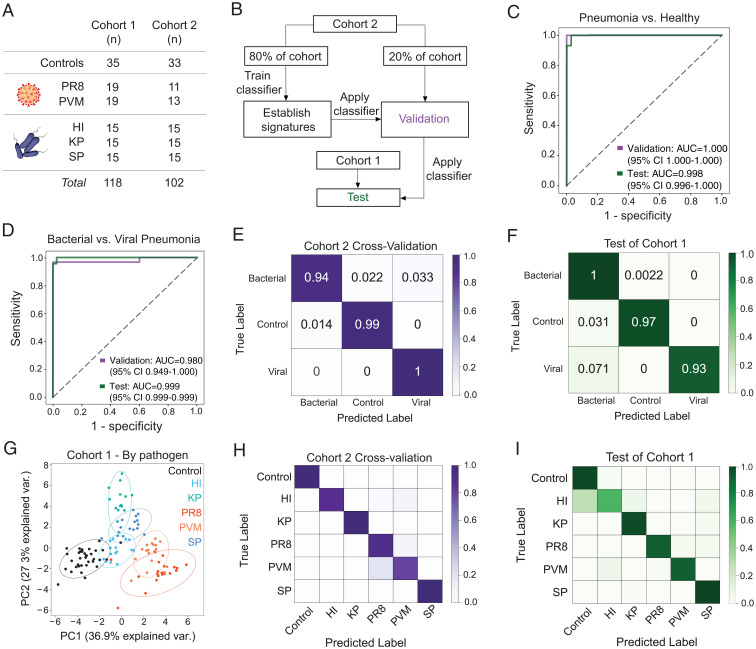
The nanosensor panel diagnoses pneumonia and classifies pneumonia etiology with high accuracy. (*A*) Mice from two independent cohorts were infected with various pneumonia-causing pathogens or given a mock dose of phosphate buffer saline for healthy controls. (*B*) Flowchart of the training, validation, and testing method used to create and test SVM classifiers for pneumonia. Cohort 2 was split into two groups: one to train the classifier and another to validate its performance. The classifier was then applied to an independent group of infected and healthy mice, cohort 1. The classification performance of the classifier on this independent cohort is labeled as the test condition. The validation and test performance of binary classifiers trained using this framework is represented with ROC curves (see *C* and *D*). Distinguishing power of classifiers trained on a multiclass prediction problem were visualized with a confusion matrix (see *E*, *F*, *H*, and *I*). (*C* and *D*) Performance of binary classifiers to differentiate mice infected with pneumonia from healthy controls (*C*) and bacterial from viral pneumonia (*D*). (*E* and *F*) Confusion matrices can visualize the performance of a multiclass SVM algorithm to distinguish among all three states of interest. The (*E*) cross-validation and (*F*) test performance of the multiclass classifier are shown here. Each value represents the frequency at which each true label was classified with the predicted label (e.g., the top left box represents the mice with bacterial pneumonia, the true label, that were classified as having bacterial pneumonia, the predicted label). The diagonal represents the true positive classifications. (*G*) PCA was performed on mean normalized urinary ABN signals from healthy controls (black) and mice with pneumonia (colored symbols) in cohort 1. (*H* and *I*) Confusion matrices showing the accuracy of an SVM classifier in pathogen identification. All performance metrics are averages over 10 independent train-test trials. Train, validation, and test *n* can be found in *Materials and Methods*.

Ultimately, our goal is to use the protease activity sensor panel to noninvasively diagnose pneumonia and simultaneously determine its etiology. Our enrichment analysis demonstrated that ABNs were differentially cleaved based on disease state (Fig. 3), and our AZP results supported that the reporters present in the urine reflect protease activity driven by pathways that are key to disease pathogenesis (Fig. 5). Having shown that the urinary reporters were reflective of disease, we next leveraged their measurements to create a diagnostic tool. For this purpose, we built a machine learning algorithm to create classifiers that could be prospectively applied to enable pneumonia diagnosis. To train the classifiers, we infected a completely new set of mice (cohort 2, *n* = 102). These mice were infected with the same pathogens as the original cohort (cohort 1, *n* = 118), but all aspects of the infection, ABN administration, and urine collection processes were performed independently (Fig. 6*A*). We then split cohort 2 into two groups: one group (*n* = 81) was used to train a support vector machine (SVM) classifier, and a second group (*n* = 21) was used to validate the classifier’s performance (Fig. 6*B*). The classifier was able to perfectly distinguish between mice that had pneumonia and healthy controls within this validation group (AUC = 1.0; Fig. 6*C*). We applied this classifier to the urinary reporter concentrations from cohort 1 to evaluate its diagnostic potential in an independent test set and achieved near-perfect classification (AUC = 0.998; Fig. 6*C*), thus demonstrating that the ABN panel can be used to design and deploy a machine learning classifier to diagnose pneumonia.

Using the same framework, we then used the reporter concentrations from the infected population of cohort 2 (*n* = 69) to train and validate a new binary classifier to distinguish between viral and bacterial pneumonia. Again, we observed very high performance in the validation cohort (AUC = 0.980; Fig. 6*D*). To test the bacterial-viral classifier, we applied it to the infected mice from cohort 1 (*n* = 83) and observed near-perfect classification of the infected mice based on pneumonia etiology (AUC = 0.999; Fig. 6*D*). After determining that separate binary classifiers could identify pneumonia and distinguish its cause, we put these capabilities together in a multiclass SVM algorithm. This multiclass differentiated among all three states (healthy, ill with bacterial pneumonia, and ill with viral pneumonia) with near-perfect accuracy (Fig. 6 *E* and *F*). These results demonstrate that our ABN platform can be used to not only identify pneumonia, but also to stratify etiology.

After establishing that machine learning classifiers, trained on our urinary reporter data, could accurately differentiate between viral and bacterial pneumonia, we next sought to evaluate whether this approach could enable pathogen-specific identification. PCA of cohort 1 showed that samples from mice infected with various pathogens formed distinct clusters (Fig. 6*G*). By training a multiclass classifier on the task of pathogen identification, we demonstrated that this classifier correctly identified the pneumonia-causing pathogen, or lack thereof in the case of the healthy controls, in each mouse with high accuracy (Fig. 6 *H* and *I*). Applying this classifier to the test set yielded highly accurate identification of all pathogens except HI, which was frequently mislabeled as healthy (Fig. 6*I*). Given these considerations, while the ABN panel can achieve pathogen identification, further optimization is required to reach a level of accuracy that would enable microbe-specific treatment.

### Multiplexing Enables Disease-Specific Classification with a Smaller Nanosensor Panel.

The complexity and heterogeneity of human disease will likely necessitate a wide panel of multiplexed ABNs that can reflect subtle differences in protease activity not only between pneumonia etiology, but also among comorbidities across varied individuals. This makes the 20-plex panel a valuable tool toward clinical translation. However, to make ABNs a viable point-of-care tool for diagnosing pneumonia in low-resource settings, we sought to determine the minimal subset of ABNs that could still achieve high classification, as a simplified panel of reporters could enable testing with lower-cost readouts such as lateral flow assays (LFAs). Based on our in situ results, we first evaluated whether the GZMB-sensitive sensors (BV01 and BV14) could be used to distinguish disease. Analysis of the full panel data revealed that this pair of ABNs is sufficient to differentiate between bacterial and viral pneumonia with high accuracy (validation AUC = 0.938, testing AUC = 0.914; *SI Appendix*, Fig. S7) when using binary classification. However, using a multiclass classifier, which resembles the clinical use case of this diagnostic, the performance of this pair did not accurately discriminate between disease states (*SI Appendix*, Fig. S7). Therefore, limiting the panel to these two ABNs may be useful for true-positive identification of viral pneumonia but would be insufficient to accurately stratify bacterial pneumonia and healthy controls.

Looking beyond GZMB, we went back to our differential enrichment analysis (Fig. 3*C*) and assessed the ability of the most differential reporters to discriminate disease. Creating a binary classifier using the top two differentially expressed reporters between bacterial and viral pneumonia (BV03 and BV19) yielded high classification of the two pneumonia etiologies (testing AUC = 0.951; *SI Appendix*, Fig. S8). However, like pairing BV01 and BV14, a multiclass classifier composed of BV03 and BV19 did not classify etiology in the wider context including healthy controls, making the pair unsuitable for use in the target clinical setting. Nonetheless, when the panel was expanded to a 5-plex of other top hits (BV19, BV03, BV13, BV09, and BV07), this set of five ABNs determined etiology with high accuracy using both binary and multiclass classifiers (*SI Appendix*, Fig. S8).

## Discussion

In this paper, we have created and validated a urine-based test that leverages the body’s response to infection to not only detect active pneumonia in mice, but also distinguish between bacterial and viral pneumonia etiologies. This diagnostic is powered by noninvasive measurements of host-derived protease activity, a means of pneumonia classification distinct from the oft-cited biomarkers such as CRP, PCT, and inflammatory cytokines. Our results reveal several insights into the diagnosis of CAP. First, we demonstrated that proteases can be mined from existing human transcriptomic data and then leveraged as biomarkers for distinguishing pneumonia etiology. Second, we created a nanosensor panel that can detect the differential activity of these proteases during pneumonia and release renally cleared reporters to generate disease-specific signatures in urine. Third, we identified a protease activity sensor, BV01, that contributes to etiology stratification by detecting the activity of GZMB, which is expressed at higher levels in a mouse model of viral pneumonia than bacterial pneumonia. The diagnostic power of this sensor appears to be driven by the greater influx of GZMB-producing immune cells into the lungs of mice in response to influenza pneumonia versus infection with SP. This observation suggests a mechanism of nanosensor activation that is dependent on differential host immunity to bacterial versus viral etiologies. Finally, we used our urinary reporter signatures to design and deploy machine learning algorithms that can diagnose pneumonia and differentiate between bacterial and viral pneumonia with high accuracy. By focusing on the proteases implicated in host immunity, we have demonstrated that pneumonia diagnostics can leverage the body’s innate response to pathogens to create noninvasive readouts of infection that are sensitive and specific.

The world’s response to the COVID-19 pandemic illustrated that rapidly determining the etiology of pneumonia is crucial for individual patient management and public health. We believe that our ABN panel represents a method to diagnose pneumonia that could augment the current diagnostic paradigm. Because sample collection for our method simply requires a urine sample, reporter analysis could likely be run in parallel with existing UATs for *Legionella* and pneumococcus, which would allow clinicians to leverage existing noninvasive tests to diagnose their patients more accurately. Furthermore, the ability of our panel to detect pneumonia and determine etiology via a noninvasive readout within 2 h of sensor administration could represent a rapid test for pneumonia. Additionally, the ability to determine pneumonia etiology quickly and accurately could help curb rising antimicrobial resistance by ruling out bacterial pneumonia on a time scale that would enable antibiotic stewardship in patients with suspected CAP.

While more work is needed to establish the potential of our approach for diagnosis of human disease, there exists a path to clinical approval and translation. The safety of the base formulation (i.e., a PEG scaffold with attached peptide linkers and conjugated mass barcodes) has already been established in both small and large animal models, as well as in healthy human subjects after intravenous administration in a phase I clinical trial (53). Furthermore, our laboratory is developing aerosolized formulations of ABNs that are suitable for pulmonary delivery in patients. Finally, we have previously developed LFAs that can detect urinary ABN reporters in paper diagnostic formats (54, 55). These advances in safety, administration, and detection establish a path to develop our activity-based diagnostic platform for clinical use.

However, beyond the clinical barriers to bringing our proof-of-principle detection approach from bench to bedside, an important biological limitation of this work is that a wide range of bacterial and viral pathogens cause CAP. Thus, to use the ABN panel as a point-of-care diagnostic, the classifier will need to be trained on urinary reporter data derived from human subjects infected with pathogens beyond the five included in this work. For example, emerging causes of CAP, such as SARS-CoV-2, should be included in datasets used to build and test diagnostic classifiers. Promisingly, recent work has shown that respiratory viruses such as influenza, SARS-CoV-2, and Ebola, among others, elicit well-conserved patterns of host immune response dysregulation (56), which supports that our diagnostic may be generalizable to novel and evolving viruses.

Another limitation of our work is that all in vivo studies were performed on female mice, but there are gender differences in CAP: namely, increased severity and mortality in males compared to females (57, 58). Such sexual dimorphism could not be captured by our urinary signatures but may be evident in clinical translation of the ABNs. However, we suspect that by multiplexing with 20 different sensors and training the classifier with urinary samples across genders, the resulting classifiers will provide coverage for any differences that may arise.

A critical step to translation is to account for the complexity of translating from animal disease models to humans. For example, there are likely differences in the cleavage patterns between the mouse and human protease homologs that would necessitate tweaking of the peptide sequences comprising the ABNs for human use (59). However, our transcriptomic signature was derived from human patients, and our in vitro screen was performed with recombinant human proteases, which makes it likely that the ABN panel will translate to humans and perhaps perform even better after optimizing the substrates to any preferred homologs. Differences in urinary reporter signatures between model organisms and humans could also be addressed by developing future ABN panels that respond to proteases produced by the pathogens themselves, as their expression and subsequent activity would be independent from the host. In this work we did not target microbial proteases, as our goal was to broadly distinguish between etiology, but the inclusion of ABNs that target microbial proteases in future nanosensor panels could enable pathogen-specific detection.

Overall, our intrapulmonary ABNs can diagnose pneumonia and distinguish etiology in several mouse models of bacterial and viral pneumonia. We have created, screened, tested, and validated the sensors using in silico, in vitro, in vivo, and in situ methods, setting the stage for further development that might one day translate into a clinically useful diagnostic for the rapid and noninvasive detection of CAP.

## Materials and Methods

### Derivation of the Protease Transcriptomic Signature.

We performed a systematic search in NIH Gene Expression Omnibus and European Bioinformatics Institute ArrayExpress for public human microarray genome-wide expression studies of tuberculosis or other diseases (60, 61). Datasets were excluded if they 1) were nonclinical, 2) were profiled using tissues other than whole blood or PBMCs, 3) did not have at least three healthy samples, or 4) did not provide information to identify whether a patient had bacterial or viral infection. We conormalized data using COCONUT (combat conormalization using controls) (11). COCONUT allows for conormalization of expression data without changing the distribution of genes between studies and without any bias toward sample diagnosis. We then applied MANATEE, which is a multicohort analysis framework that is used to integrate gene expression datasets, perform differential expression analyses to filter out top genes, apply machine learning methods to arrive at a concise diagnostic signature, and finally to validate the discovered signature in independent data (Fig. 2*A*) (27). Details about each of these steps can be found in *SI Appendix*, *Supplemental Methods*.

### Animal Models.

All animal studies were approved by the MIT Institutional Animal Care and Use Committee (protocol 0619-032-44) and were conducted in compliance with institutional and national policies. The 7- to 9-wk-old female mice (BALB/c, Taconic) were dosed with either SP (NCTC 7466), KP (ATCC 43816), HI (ATCC 33391), PVM (ATCC VR-1819), or influenza (Influenza A/PR/8/34 (H1N1), Charles River). Nanosensors were synthesized by CPC Scientific. ABNs were dosed in mannitol buffer (0.28 M mannitol, 5 mM sodium phosphate monobasic, 15 mM sodium phosphate dibasic, pH 7.0 to 7.5) and deposited into the lungs by intratracheal instillation (50 µL total volume, 20 µM per ABN). After 1 h, the bladder was manually voided, the urine was discarded, and the mice were put into a collection chamber for the next hour. Two hours after ABN administration, the bladder was manually voided and the urine was collected, along with any urine that was produced in the collection chamber. These urine samples were then sent to Syneos Health for liquid chromatography-tandem mass spectrometry (LC-MS/MS) analysis. Reporter quantification by LC-MS/MS was performed as previously described (26).

### Statistical Analysis.

For in vivo analysis, PCA, reporter enrichment, and the SVM algorithm training/validation were performed using our group’s Protease Activity Analysis toolkit, a publicly available Python package designed to process and visualize enzymatic activity datasets (62). Transcriptomic datasets and the in vitro protease screen hierarchical clustering were analyzed in R (https://www.r-project.org/). All other analyses were performed in GraphPad 9.0 (Prism). For disease classification based on urinary ABN signatures, randomly assigned sets of paired data samples consisting of features (i.e., standardized scores of peak area ratio of individual urinary reporters measured by LC-MS/MS) and labels (i.e., bacterial or viral) were used to train linear SVM classifiers implemented in Python 3. All analyses were run with 10-fold cross-validation, and trained classifiers were tested on randomly assigned, held-out, independent test cohorts. Classification performance was evaluated with ROC statistics. Classifier performance was reported as the mean accuracy and AUC across 10-fold independent cross-validations.

## Supplementary Material

Supplementary File

## Data Availability

All materials are included in the manuscript and/or *SI Appendix*. The code used to analyze the in vitro and in vivo data have been deposited into publicly available repositories. The MANATEE scripts associated with this paper can be found at GitHub: https://github.com/Khatri-Lab/manatee_pnas. The code used for analysis of the in vivo data can be accessed by downloading the Protease Activity Analysis toolkit at GitHub (https://github.com/apsoleimany/protease_activity_analysis).
