# Supplementary material for: Host protease activity classifies pneumonia etiology
Source: Proc Natl Acad Sci U S A. 2022 Jun 13;119(25):e2121778119. doi: 10.1073/pnas.2121778119 (PMC9231472; doi:10.1073/pnas.2121778119)
Supplement: Supplementary File [file pnas.2121778119.sapp.pdf]

PNAS
www.pnas.org

Supplementary Information for
Host Protease Activity Classifies Pneumonia Etiology

Melodi Anahtar, Leslie W. Chan, Henry Ko, Aditya Rao, Ava P. Soleimany, Purvesh Khatri*, Sangeeta N. Bhatia*

Purvesh Khatri, Sangeeta N. Bhatia
Email: sbhatia@mit.edu

This PDF file includes:

- Supplemental Methods
- Supplemental References
- Figures S1 to S8
- Tables S1 to S4

Supplemental Methods

Systematic search for gene expression datasets

We performed a systematic search in NIH Gene Expression Omnibus (GEO) and European Bioinformatics Institute (EBI) ArrayExpress for public human microarray genome-wide expression studies of TB or other diseases (1, 2). Datasets were excluded if they (i) were nonclinical, (ii) were profiled using tissues other than WB or PBMCs, (iii) did not have at least 3 healthy samples, or (iv) did not provide information to identify whether a patient had bacterial or viral infection.

All microarray data were renormalized from raw data (when available) using standardized methods. Affymetrix arrays were renormalized using GC robust multiarray average (gcRMA) (on arrays with mismatch probes) or RMA. Illumina, Agilent, GE, and other commercial arrays were renormalized via normal-exponential background correction followed by quantile normalization. Custom arrays were not renormalized. Data were log2-transformed, and a fixed-effect model was used to summarize probes to genes within each study. Within each study, cohorts assayed with different microarray types were treated as independent.

COCONUT conormalization

We conormalized data using COCONUT or Combat CONormalization Using conTrols (3). COCONUT allows for conormalization of expression data without changing the distribution of genes between studies and without any bias towards sample diagnosis. It applies a modified version of the ComBat empirical Bayes normalization method that only assumes an equal distribution between control samples (4). Briefly, the healthy controls from each cohort undergo ComBat conormalization without covariates, and the ComBat estimated parameters are acquired for each dataset's healthy samples. These parameters are then applied to the diseased samples in each dataset, which causes all samples to assume the same background distribution while still retaining the relative distance between healthy and diseased samples in each dataset. We have previously shown that when COCONUT conormalization is applied, housekeeping genes remain invariant

across both diseases and cohorts, and each gene still retained the same distribution between diseases and controls within each data set.

Derivation of the 39-protease signature with MANATEE

MANATEE or Multicohort ANalysis with AggregaTed gEne Expression is a multicohort analysis framework that is used to integrate gene expression datasets, perform differential expression analyses to filter out top genes, apply machine learning methods to arrive at a concise diagnostic signature, and finally to validate the discovered signature in independent data (**Figure 2A**) (5). In this analysis, any genes that did not code for proteases were removed from all datasets. Next, relevant datasets were identified through a systemic search of public gene expression data repositories. Some of these datasets were chosen for training the signature, and the rest were set aside as future independent validation datasets. Samples from the training datasets were then randomly split, with 70% of the samples assigned to Discovery and the other 30% assigned to Hold-out Validation. The Discovery and Hold-out Validation cohorts were each batch corrected with COCONUT conormalization.

Next, differential expression statistics were calculated in Discovery. Here, we computed four measures of differential expression between cases and controls are calculated for each protease: (1) the SAM score (from the Significance Analysis of Microarrays or SAM method (*33*), (2) the corresponding SAM local FDR, (3) the Benjamini-Hochberg FDR corrected P value (from running a t-test, (*34*) and (4) the effect size (ES). The effect size is estimated as Hedges' adjusted g, which accounts for small sample bias (6–9). We also performed a leave-one-study-out (LOSO) analysis, wherein each study that accounted for at least 5% of the training samples was iteratively removed from the training set, and the differential expression statistics were re-calculated for each version of the training set with one study left out. Thus, in order for a protease to be selected, it must not only exceed the given thresholds in the statistics calculated for the full training set, but it must also exceed those thresholds for each version of the training set with one study removed. This prevents any single study from exerting too strong of an effect on the selection of proteases. (10, 11)

Once the differential statistics were calculated, a set of “top” differentially expressed proteases was chosen by filtering out proteases that had an FDR of < 0.01 and an absolute effect size of > 0.6. This resulted in a 39-protease signature. The signature was first tested in Hold-out Validation to assess whether the signature’s performance remained robust when tested in new data. Finally, the signature was tested in Independent Validation to measure its performance in completely independent data. The MANATEE scripts associated with this paper are publicly available and can be found at the following repository: https://github.com/Khatri-Lab/manatee_pnas.

Enrichment analysis with ConsensusPathDB

The bacterial and viral gene signatures were input into ConsensusPathDB for over-representation analysis. For the pathway analysis there was a minimum overlap of 3 candidates and a p-value cutoff < 0.01. The entity graph visualization was performed using the database and edges with no shared candidates between nodes were filtered out.

Recombinant substrate screens with fluorescent substrates

Quenched fluorogenic probes were synthesized by CPC Scientific (sequences in **Table S3**). Each probe was diluted first in dimethylformamide (DMF), subsequently in PBS, and plated into a 384-well plate. The plates were sealed and stored at -20°C until needed. To perform the cleavage assay, recombinant proteases were activated as necessary and diluted in their respective assay buffers with 0.1% BSA. The recombinant proteases were then added to each substrate containing well for a final reaction volume of 50 µL (20 µM substrate and 20 nM recombinant protease per well). Control wells, which contained no protease, were run on the same plate. Each protease-substrate pair and relevant blank control was plated in duplicate. Cleavage over time was quantified by fluorescence as measured by a fluorimeter (Tecan Infinite M200 Pro). Fold change was calculated as the fluorescent signal at 10 minutes divided by the original fluorescence at the start of the read. All enzyme sources and buffers can be found in **Table S4.**

Mouse pneumonia models

All animal studies were approved by the MIT IUCAC (protocol 0619-032-44) and were conducted in compliance with institutional and national policies. 7- to 9-week-old female mice (BALB/c, Taconic) were dosed with either *S. pneumoniae* (NCTC 7466), *K. pneumoniae* (ATCC 43816), *H. influenzae* (ATCC 33391), pneumonia virus of mice (ATCC VR-1819), or influenza (Influenza A/PR/8/34 (H1N1), Charles River). The infectious dose for each pathogen was selected based on physical signs of infection in the mice and plated colony counts (for bacteria) (see **Figure S1**). To administer the pathogens, mice were first anesthetized by isoflurane inhalation (Zoetis). While under anesthesia, pathogens were passively inhaled via either intratracheal instillation (IT, for *S. pneumoniae, K. pneumoniae,* and *H. influenzae*) or intranasally (for PVM and Influenza A). A volume of 50 µL was administered for all pathogens except Influenza A, which was administered at 30 µL. Age- and gender-matched control mice in each experiment received either 50 µL of sterile-filtered PBS IT for the bacterial cohorts or IN for the viral cohorts.

Pathogen preparation

To prepare the bacteria, all bacteria were first cultured overnight (37°C, shaking at 250 rpm for 14-20 hours) and subsequently grown in secondary culture with 1:100 to 1:200 dilutions to an OD600 of 0.5-0.7, corresponding to a phase of exponential growth. *K. pneumoniae* was cultured in LB broth (Invitrogen). *S. pneumoniae* was plated overnight on blood-agar plates with neomycin (Hardy Diagnostics), and subsequently cultured in liquid brain-heart infusion (BHI; BD) media. *H. influenzae* was cultured in supplemented BHI (BHI with NAD and histidine-hemin). They were then pelleted, washed three times with sterile-filtered PBS and diluted to the appropriate concentration for administration. To prepare the viruses for infection, all viruses were diluted directly into sterile-filtered PBS from aliquoted stocks and kept on ice until administration.

qRT-PCR for viral loads and GZMB

Lungs were dissected from infected and healthy mice, rinsed in PBS and stored in RNAlater (Sigma Aldrich) at -80°C until use. RNA extraction was performed using the RNeasy Mini

kit (Qiagen). On-column DNase digestion was performed using the RNase-Free DNase Set (Qiagen). RNA concentration was measured on a Nanodrop at A260. cDNA was prepared with the RevertAid First Strand cDNA synthesis kit (Thermo Fisher). qRT-PCR was performed using Ssofast EvaGreen Supermix (Bio-Rad). For viral load quantification, custom oligo primers for PR8 and PVM were ordered from IDT (PR8: *PA* gene, Forward: 5' GCG GTC CAA ATT CCT GCT GA 3', Reverse: 5' CAT TGG GTT CCT TCC ATC CAA AG 3'; PVM: *SH* gene, Forward: 5' GCC GTC ATC AAC ACAG TGT GT 3', Reverse: 5' GCC TGA TGT GGC AGT GCT T 3'). Viral loads were estimated by running a standard curve with custom gBlocks from IDT for PR8 (*PA* gene) and PVM (*SH* gene). Relative Granzyme B expression was measured using custom primers (IDT, Forward: 5' TCT CTG ACT CCA CGT CTC TTA C 3', Reverse: 5' CTG GGT CTT CTC CTG TTC TTT G 3'). GAPDH expression was measured for normalization (ReadyMade primers, IDT).

In vivo activity-based nanosensor studies

Nanosensors were synthesized by CPC Scientific. ABNs were dosed in mannitol buffer (0.28 M mannitol, 5 mM sodium phosphate monobasic, 15 mM sodium phosphate dibasic, pH 7.0-7.5) and deposited into the lungs by intratracheal instillation (50 μL total volume, 20 μM per ABN). Immediately after dosing, all mice were given a subcutaneous injection of PBS (400 μL) to promote adequate urine volumes for subsequent analysis. For the viral pneumonia models, mice were administered the ABN cocktail 6 days post infection (p.i.). For the bacterial pneumonia models, mice were administered ABNs 16 hours p.i. For all mice, after receiving the ABNs mice were returned to their home cage for one hour with full access to food and water. After this hour their bladder was manually voided, and they were transferred into a urine collection chamber. At the end of the second hour, the bladder was manually voided and the urine was collected, along with any urine that was produced in the collection chamber. The urine samples were then sent to Syneos Health for LC-MS/MS analysis. Reporter quantification by LC-MS/MS was performed as previously described (12).

At least 15 mice were infected with each pathogen, alongside at least 5 healthy control mice per pathogen. These sample sizes were chosen to ensure that each group would be greater

than or equal to ten in order to properly train and test the diagnostic classifiers. After infection, mice were monitored daily. In the first cohort, one mouse from each viral model died before urine collection, and in cohort 2 several urine samples had volumes that were too low for analysis with mass spectrometry. Aside from these natural losses, no samples were excluded from analysis. Healthy and infected mice were caged separately to prevent any possible cross-contamination. Investigators were not blind to infection status as proper decontamination and sterility measures needed to be taken to minimize cross-contamination and ensure safety of the researchers.

Tissue dissection from mice and slide preparation

Female BALB/c mice were infected with influenza A (PR8) or *S. pneumoniae* (SP) as described above. PR8 and SP mice were euthanized at 6 days and 16 hours after infection initiation, respectively. The lungs were removed from the infected mice or healthy controls, and put into a 6-well plate filled with PBS while the lobes were separated. The individual lobes were then immediately embedded in optimal-cutting-temperature (OCT) compound (Sakura), frozen in isopentane chilled with dry ice, and stored at -80ºC until sectioning. Cryosectioning was performed at the Koch Institute Histology Core. The resulting slides were then stored at -80ºC until use for immunofluorescent staining or AZP experiments.

Immunofluorescent staining for immune cell markers and GZMB

Fresh-frozen slides were prepared as described above. To prepare the slides for staining, they were air-dried for 20 minutes, fixed in ice-cold acetone for 10 minutes, air-dried for 20 minutes and washed in sterile PBS (3x5 minutes). After the final wash, the tissue sections on each slide were outlined with an ImmEdge Pen (Vector Laboratories Inc, Burlingame, CA) and blocked with 1% BSA in PBS for 30-45 minutes. The blocking buffer was then aspirated and replaced with the relevant primary antibodies in PBS (Granzyme B, Abcam 25598, 2.93 ug/mL; RB6-8C5, Abcam 25377, 5 ug/mL; Mouse NKp46/NCR1 Antibody, R&D AF2225, 4 ug/mL; CD8 (53-6.7), Novus NBP1-49045, 5 ug/mL), and incubated at room temperature for 1.5 hours. Slides were washed with PBS (3x5 minutes) and incubated with appropriate secondary antibodies and Hoechst (1:2000

dilution) for 30 minutes at room temperature. They were then washed (3x5 minutes) and mounted (ProLong Diamond Antifade Mountant, Invitrogen).

In situ zymography with AZPs

For experiments involving on-slide AZP activation, slides were dried and fixed as previously described. Slides were blocked with either Granzyme B inhibitor (Z-AAD-CH2Cl, Abcam ab142034) with 1% BSA and 100 μM inhibitor in PBS, a protease inhibitor cocktail (Halt™ Protease Inhibitor Cocktail, diluted 1:100, with additional 400 uM AEBSF and 1 mM Marimastat) with 1% BSA, or no inhibitors with 1% BSA and an equivalent volume of DMSO to the inhibitors. After blocking, slides were incubated with BV01-Z (1 μM), Cy7-polyR (1 nM), and either GZMB inhibitor (100 μM), a protease inhibitor cocktail (Halt™ Protease Inhibitor Cocktail, diluted 1:100, with additional 400 uM AEBSF and 1 mM Marimastat) or an equivalent DMSO volume diluted in buffer (50 mM Tris, pH 7.5) for 2 hours at 37ºC. Slides were washed with PBS (3x5 minutes) and incubated with Hoechst (1:2000 dilution) for 10 minutes at room temperature. Slides were washed again (3x5 minutes) and mounted as previously described. For pre-cleavage experiments, recombinant human Granzyme B (R&D 2096-SE, 100 ug/mL) was activated with recombinant mouse Cathepsin C (R&D 2336-CY-010, 10 ug/mL) in activation buffer (50 mM MES, 50 mM NaCl, pH 5.5) for 4 hours at 37°C. The activated rhGZMB was diluted to 100 nM and incubated with BV01-Z (10 μM) and DTNB (100 μM) in assay buffer (50 mM Tris, pH 7.5) for 4 hours at 37°C. Meanwhile, fresh-frozen slides with healthy lung tissue were prepared and blocked with BSA as described. After blocking, the slides were either incubated at 4C for 1 hour with the pre-cleaved BV01-Z mixture or intact BV01-Z, Cy7-polyR and DTNB diluted in assay buffer. Slides were then washed (3x5 minutes), stained with Hoechst, washed, and mounted as previously described.

Quantification of immunofluorescent staining and AZP signal

All slides were imaged on a Pannoramic 250 Flash III whole slide scanner (3DHistech). Whole slide images were imported into QuPath (0.2.3) for quantification. Individual cells were detected using the cell detection feature on the DAPI channel. Intensity thresholds were manually

determined based on mean and maximum intensity distributions for each channel, in order to classify cells as being either positive or negative for any given marker (CD8, NK, GZMB, Ly6G). Using scripts, each cell on the slide was annotated based on whether it met the threshold, and the percentage of positive cells for each marker was calculated using Excel. For AZP quantification, the mean intensity of each cell in the AZP and polyR channels was calculated and the ratio of those values was interpreted as the relative AZP signal. All further statistical measurements were performed in GraphPad 9.0 (Prism).

Supplemental References

1. N. Kolesnikov, *et al.*, ArrayExpress update-simplifying data submissions. *Nucleic Acids Research* **43**, D1113–D1116 (2015).
2. R. Edgar, M. Domrachev, A. E. Lash, Gene Expression Omnibus: NCBI gene expression and hybridization array data repository. *Nucleic Acids Research* **30**, 207–210 (2002).
3. T. E. Sweeney, H. R. Wong, P. Khatri, Robust classification of bacterial and viral infections via integrated host gene expression diagnostics. *Science Translational Medicine* **8** (2016).
4. W. E. Johnson, C. Li, A. Rabinovic, Adjusting batch effects in microarray expression data using empirical Bayes methods. *Biostatistics* **8**, 118–127 (2007).
5. A. M. Rao, *et al.*, A Robust Host-Response-Based Signature Distinguishes Bacterial and Viral Infections Across Diverse Global Populations. *SSRN Electronic Journal* (2021) https:/doi.org/10.2139/SSRN.3962154 (March 9, 2022).
6. C. P. Doncaster, R. Spake, Correction for bias in meta-analysis of little-replicated studies. *Methods in Ecology and Evolution* **9**, 634–644 (2018).
7. L. v. Hedges, A random effects model for effect sizes. *Psychological Bulletin* **93**, 388–395 (1983).
8. L. v. Hedges, Estimation of effect size from a series of independent experiments. *Psychological Bulletin* **92**, 490–499 (1982).
9. D. Lakens, Calculating and reporting effect sizes to facilitate cumulative science: a practical primer for t-tests and ANOVAs. *Frontiers in Psychology* **0**, 863 (2013).
10. S. Arlot, A. Celisse, A survey of cross-validation procedures for model selection. *https://doi.org/10.1214/09-SS054* **4**, 40–79 (2010).
11. M. Stone, Cross-Validatory Choice and Assessment of Statistical Predictions. *Journal of the Royal Statistical Society: Series B (Methodological)* **36**, 111–133 (1974).
12. J. D. Kirkpatrick, *et al.*, Urinary detection of lung cancer in mice via noninvasive pulmonary protease profiling. **0262** (2020).

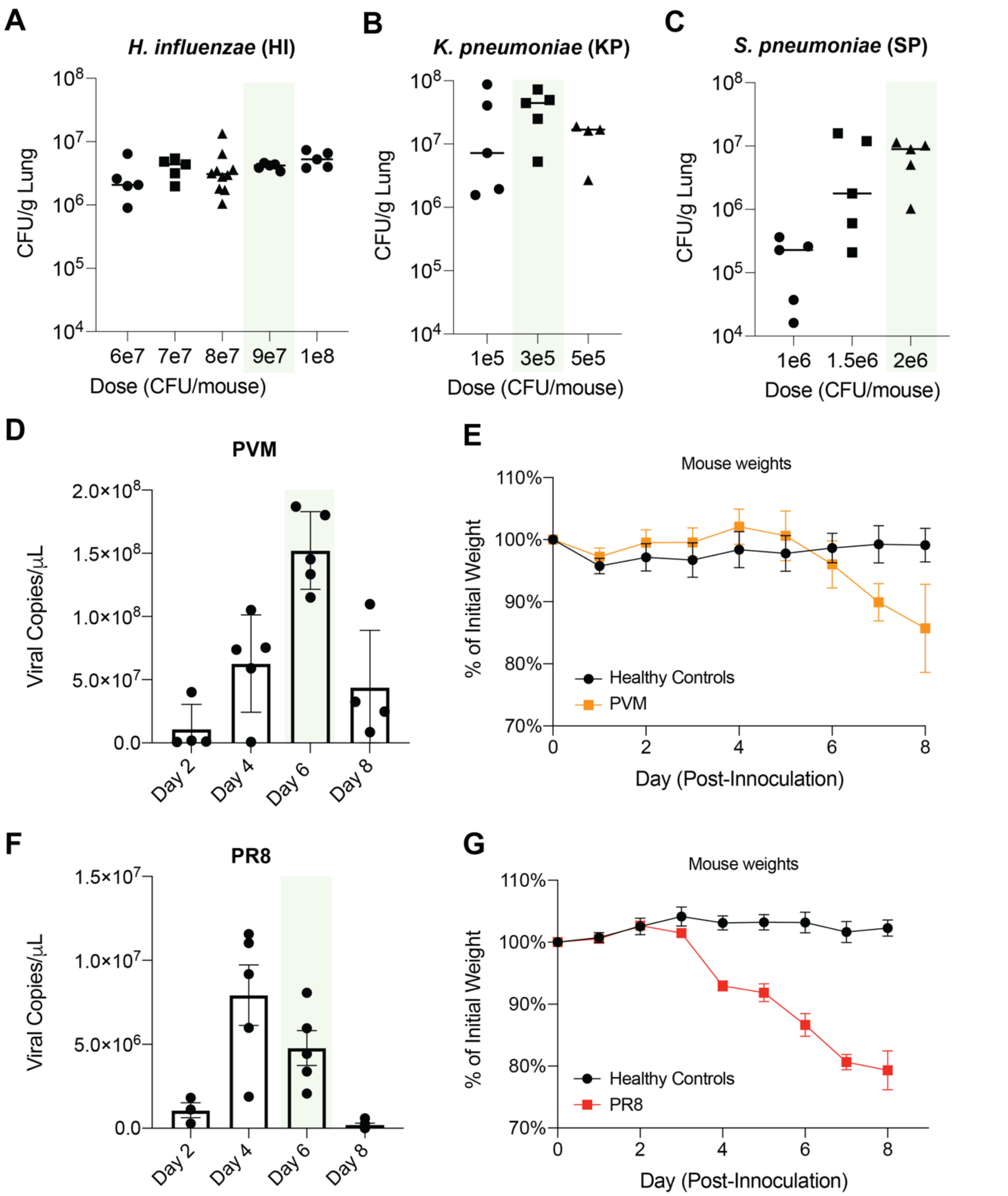

Figure S1. Characterization of the mouse models for bacterial and viral pneumonia. (A,B,C) Various doses of each bacteria were administered to immunocompetent mice. Lungs from these mice were harvested 16 hours after infection initiation, homogenized and plated to determine bacterial loads. Each point represents one mouse, n = 5 to 10 per dose. (D,F) The viral load in mice infected with pnuemonia virus of mice (PVM, 300 PFU/mouse) and influenza A (PR8, 3.8e4 EID50/mouse) was evaluated over time. Viral loads were quantified using qRT-PCR. n=3-5 mice per timepoint. (E,G) The physical manifestations of disease were tracked via body weight throughout the timecourse of infection. n=3-5 mice per timepoint. In all dose characterization graphs, green bars indicate the chosen condition (either dose or timepoint) that was used for each model.

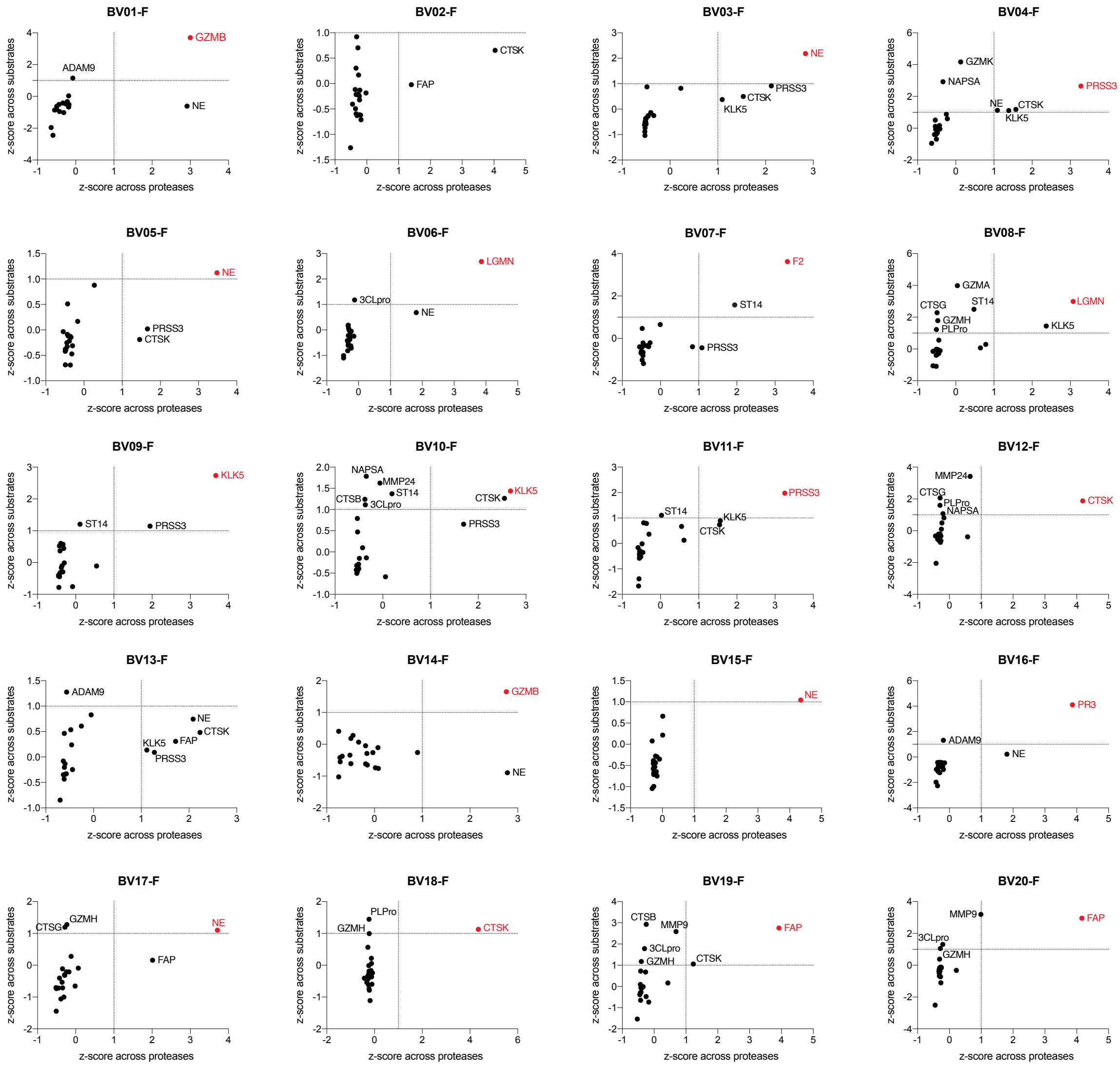

Figure S2. Specificity versus efficiency (SvE) plots can be used to identify optimal protease-substrate pairings. Each plot visualizes the correlation between standardized metrics that were calculated based on the fluorescence fold change at 10 minutes after incubation of the fluorescent probe with each recombinant protease. The x-axis plots the z-score of the fluorescent fold change across the screen proteases, which effectively quantifies specificity of each substrate for a given probe. The y-axis plots the z-score across the screened substrates, indicating how efficiently each probe was cleaved by a given protease. Dotted lines are plotted along each axis at a z-score of 1, to delineate hits that are one standard deviation or above the mean for each metric. The most protease with the highest of both metrics is in red, but other proteases in the upper rightmost quadrant are also considered “optimal” hits, and any pairing that scores above a 1 on either metric is labeled.

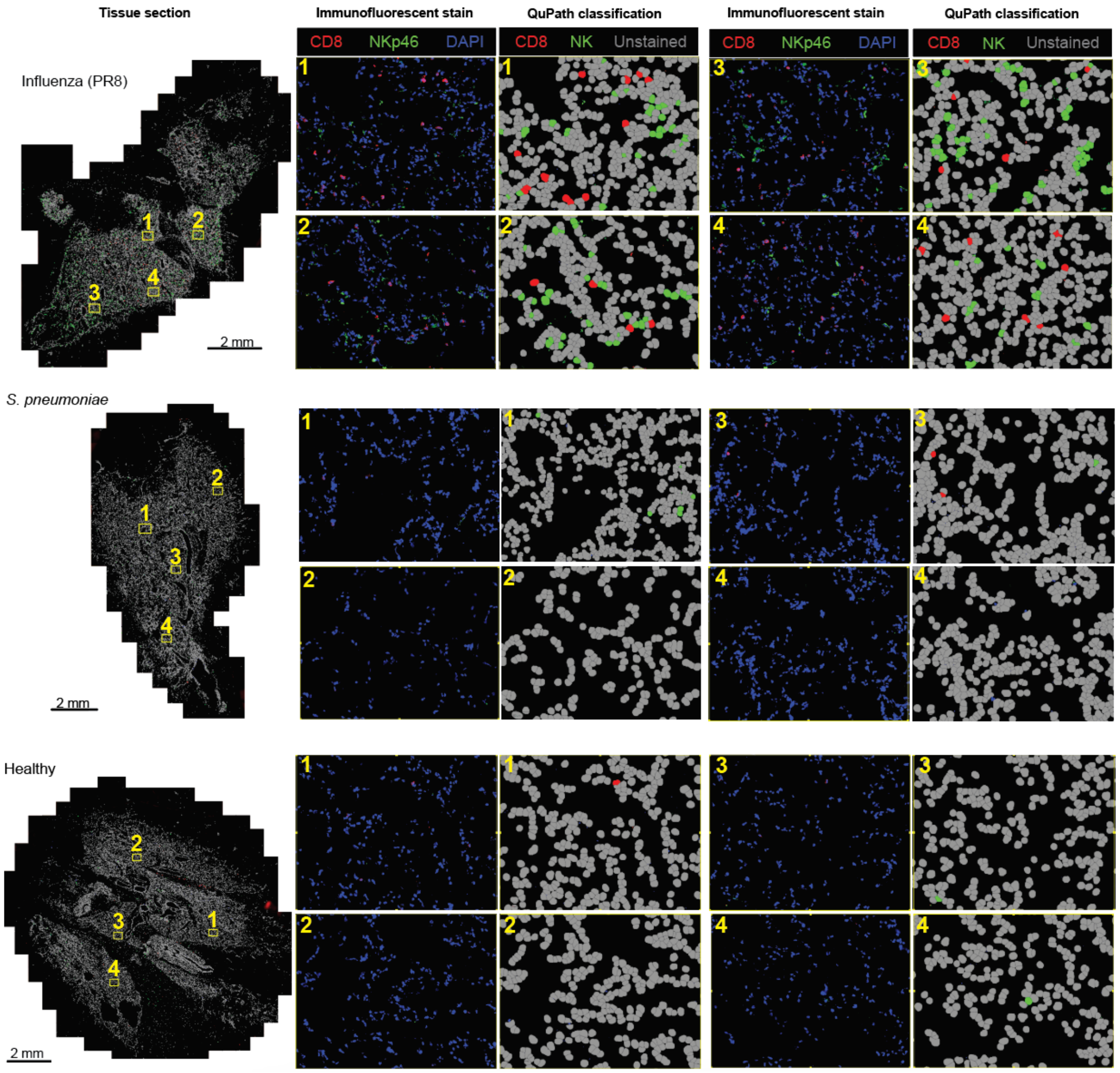

Figure S3. Immunofluorescent staining of infected lung tissue reveals differences in immune cell recruitment between *S. pneumoniae* and influenza. Representative images of viral influenza (PR8), bacterial *S. pneumoniae* (SP), and healthy lungs stained for NKp46 and CD8. Staining for each disease state was done on consecutive slides (n=2 sections/slide). (B) Based on the staining, cells were classified as either natural killer cells (NK) or CD8 T cells (CD8), and are colored green (NK) or red (CD8) if they were considered stain positive based on human determined thresholds that were kept consistent across disease states for each antibody. The colors are the result of artificial recoloring done by QuPath AF488 and Cy7 secondary antibodies, respectively. The immunofluorescent images were counterstained with DAPI (blue), which was used to automatically detect all cells present in the slide. Total cell count was used as the denominator for calculating the percentage of target staining across each slide; quantification of percent-positive cells and total cell counts can be found in Figure 5A,B.

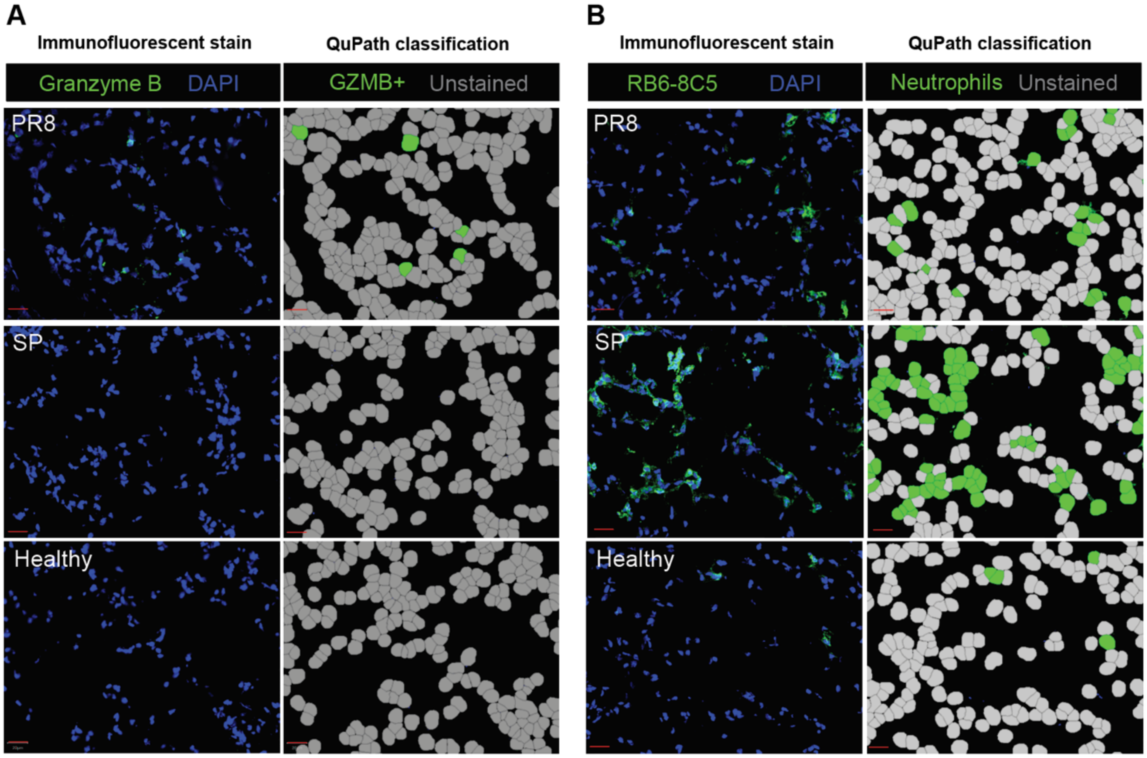

Figure S4. Immunofluorescent staining of infected lung tissue reveals differences in protease expression and neutrophil recruitment between *S. pneumoniae* and influenza. Representative images of viral influenza (PR8), bacterial *S. pneumoniae* (SP), and healthy lungs stained for Granzyme B (A) and RB6-8C5, a neutrophil marker that binds to Ly6G. Staining for each disease state was done on consecutive slides (n=2 sections/slide). Based on the staining, cells were classified as either (A) GZMB-expressing or (B) neutrophil-lineage, and are colored green (automatic recoloration by QuPath of AF488 secondary antibodies for each stain) if they were considered stain positive based on human determined thresholds that were kept consistent across disease states for each antibody. The immunofluorescent images were counterstained with DAPI (blue), which was used to automatically detect all cells present in the slide. Total cell count was used as the denominator for calculating the percentage of target staining across each slide; quantification of percent-positive cells and total cell counts can be found in Figure 5C,D. Red scale bars = 20 um.

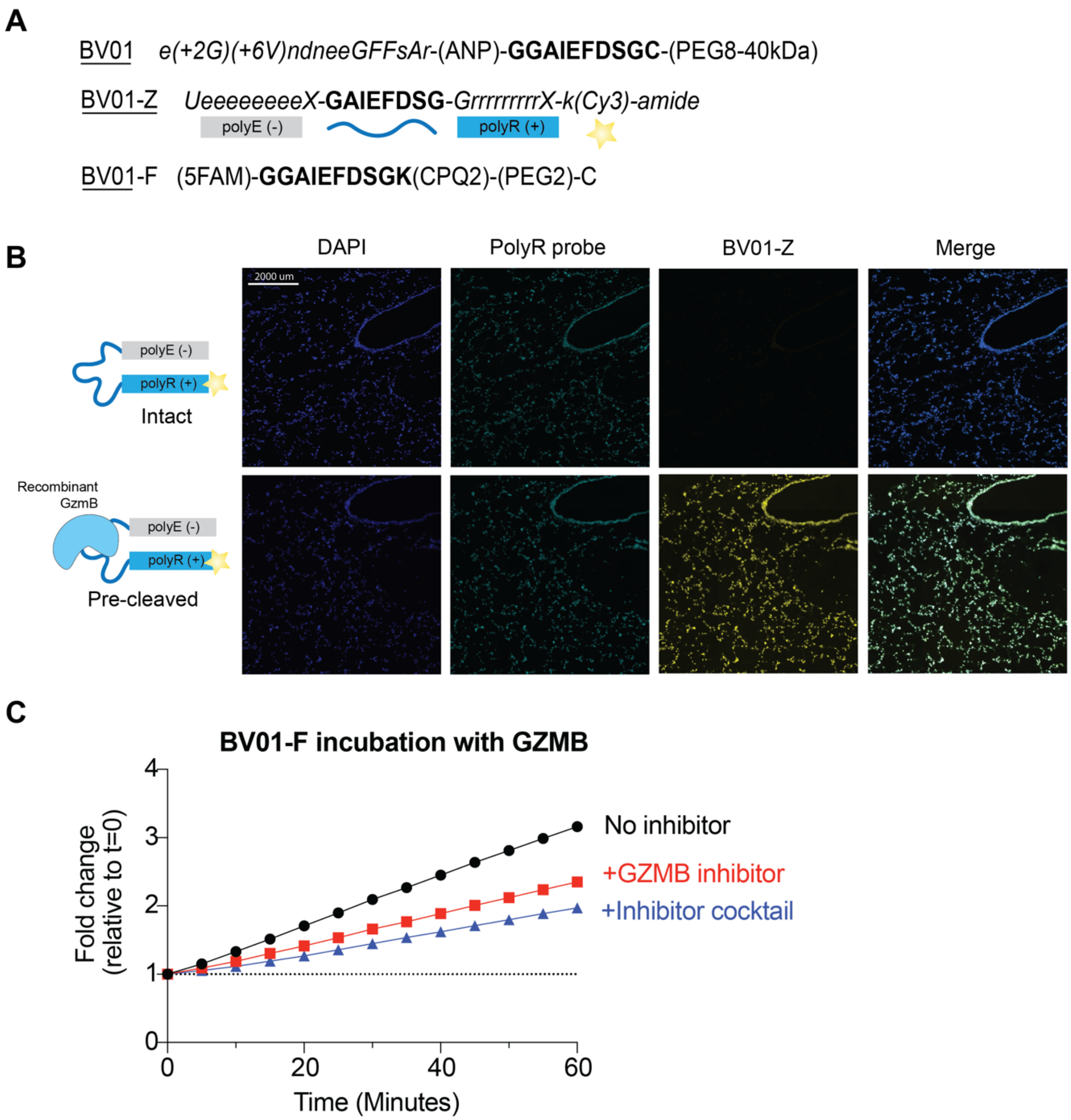

Figure S5. BV01-Z is cleaved by recombinant Granzyme B and its activity is abrogated by the addition of protease inhibitors. (A) The peptide sequence from BV01 (the ABN format) was also used to create an AZP (BV01-Z) and a fluorogenic substrate (BV01-F). (B) Staining of fresh frozen healthy lung tissue with intact probe and BV01-Z that was incubated with GZMB before being applied to tissue. Staining shows free polyR (teal) and the cleaved polyR domain (yellow). (C) Activated recombinant Granzyme B was pre-incubated with either the GZMB specific inhibitor or the protease cocktail for 3 hours at 37ºC. The resulting GZMB with and without inhibition was then incubated with BV01-F (20 uM final concentration in the well), and cleavage of BV01-F was measured over the course of 1 hour. The relative fold change in fluorescence, which reflects substrate cleavage, compared to t=0 is shown.

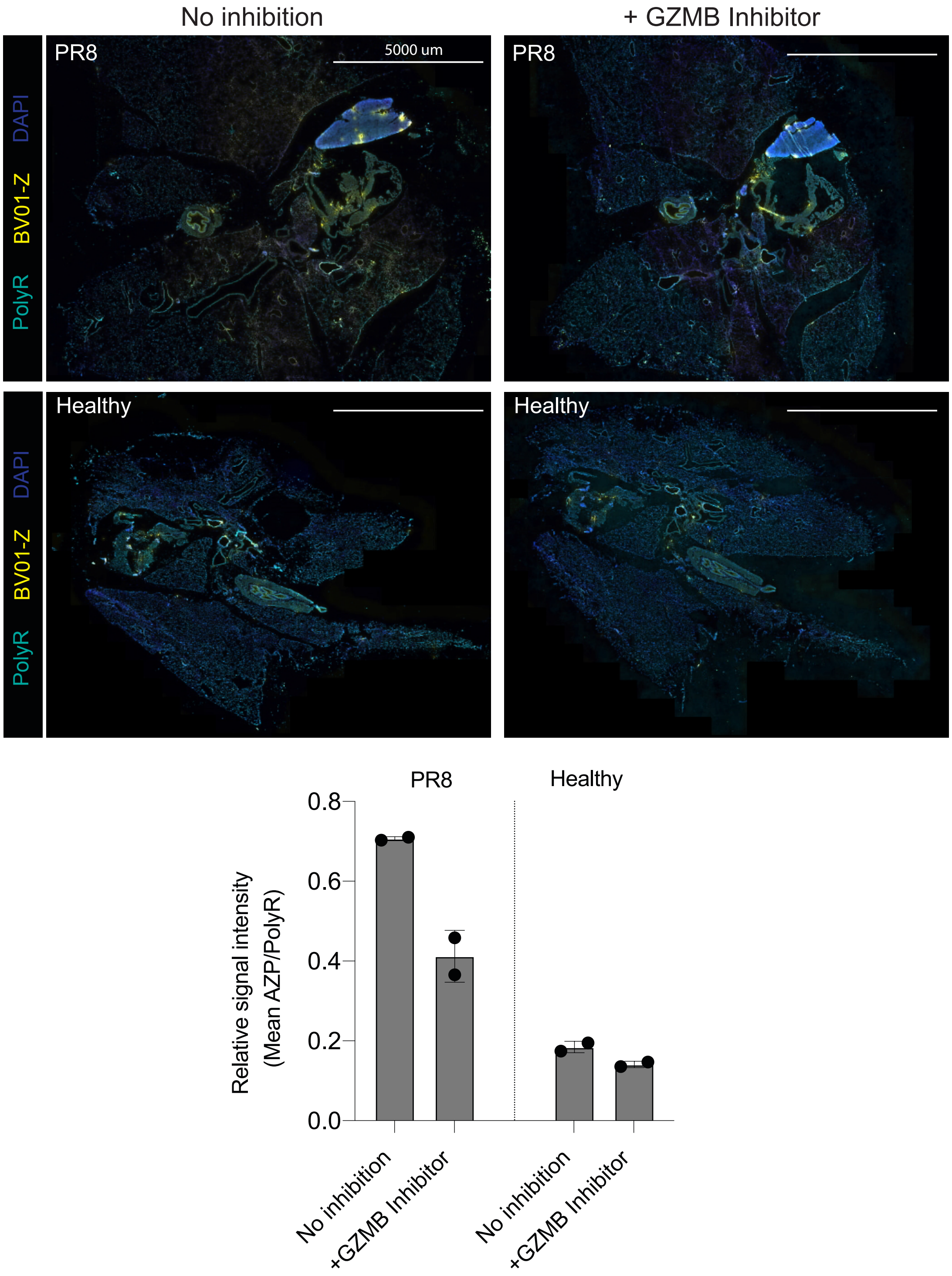

Figure S6. BV01-Z signal in mice with influenza versus healthy controls correlates with disease state and is abrogated in the presence of a Granzyme B inhibitor. Staining of fresh frozen lung from PR8 infected mice and healthy tissue with no inhibition or in the presence of a GzmB specific inhibitor. Each image is representative of consecutive sections. The ratio of the AZP signal (yellow) to free polyR (teal) is quantified (n=2 per condition). Counterstained with DAPI (blue).

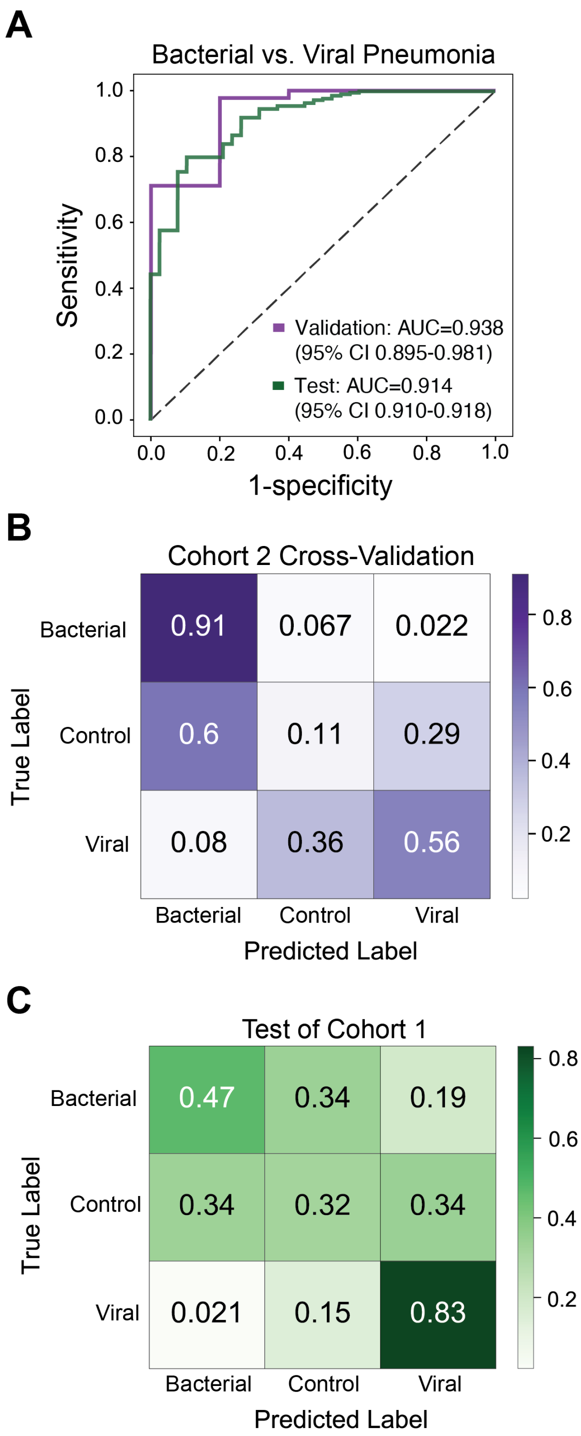

Figure S7. Classifiers can be trained using urinary reporter concentrations of GZMB specific ABNs to discriminate among disease states. (A) A binary classifier was trained using the *in vivo* concentrations of BV01 and BV14, which are both predicted to be indicative of GZMB activity, and tested to determine their diagnostic potential for discriminating bacterial and viral pneumonia. (B, C) Confusion matrices were then used to evaluate the performance of a multiclass SVM algorithm trained on BV01 and BV14 to diagnose pneumonia and stratify etiology. All classifiers are averages over 10 independent train-test trials.

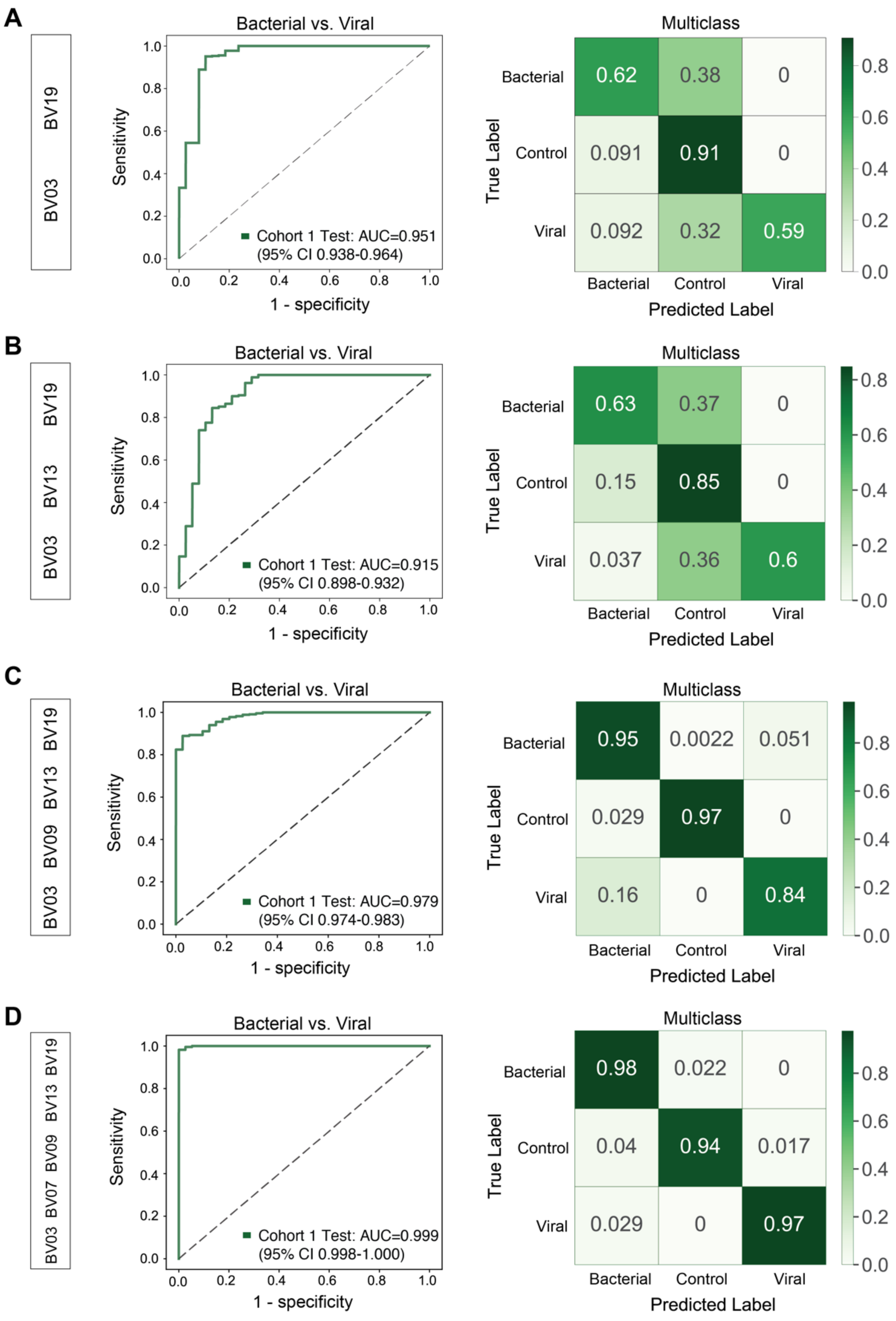

Figure S8. A subset of five ABNs can achieve high binary and multiclass classification of etiology. ROC curves and confusion matrices showing the performance of a support vector machine trained on urinary reporters from the mice in Cohort 2. Each pair of graphs shows the performance after training classifiers using the changing set of reporters listed on the left. All classifiers are averages over 10 independent train-test trials.

Table S1. Discovery datasets for creating transcriptomic signatures. Cohorts of human transcriptomic data was used to train and validate a diagnostic classifier for bacterial and viral infections. Information on the source of the data and the patient cohorts that were used to train the classifier are listed.

Accession	Author	Platform	Tissue	Location	Demographic	Bacteria	Viruses	# of healthy samples	# of bacterial samples	# of viral samples
GSE101702	Yu	GPL21185	WB	Australia, Canada, Germany	Adults with influenza		Influenza	52	0	72
GSE117827	Tang	GPL23126	WB	USA	Children with acute viral infection		HRV, RSV, Enterovirus, Coxsackievirus	6	0	18
GSE16129 GPL96	Ardura	GPL96	PBMC	USA	Children with invasive staph infections	S. aureus		10	4	0
GSE17156	Zaas	GPL571	WB	USA, UK	Adults with respiratory viral infection		Influenza, HRV, RSV	56	0	27
GSE19491	Berry	GPL6947	WB	UK, South Africa	Patients with febrile bacterial infection	S. pyogenes, Staphylococcus spp.		18	75	0
GSE20346	Parnell	GPL6947	WB	Australia	Adults with CAP	Unknown	Influenza	18	6	4
GSE21802	Bermejo-Martin	GPL6102	WB	Spain	Adults with septic influenza		Influenza	4	0	12
GSE27131	Berdal	GPL6244	WB	Norway	Adults with influenza		Influenza	7	0	7
GSE38900 GPL10558	Mejias	GPL10558	WB	USA	Children with acute LRTI		RSV	8	0	28
GSE38900 GPL6884	Mejias	GPL6884	WB	USA, Finland	Children with acute LRTI		Influenza, HRV, RSV	31	0	153
GSE40012	Parnell	GPL6947	WB	Australia, Hong Kong	Adults with CAP	Unknown	Influenza	18	16	8
GSE42026	Herberg	GPL6947	WB	UK	Children admitted with febrile infections	Gram-positive	Influenza, RSV	33	18	41
GSE60244	Suarez	GPL10558	WB	USA	Adults hospitalized with LRTI	Unknown		40	22	0
GSE64456	Mahajan	GPL10558	WB	USA	Febrile infants ≤ 60 days old		Influenza, RSV, Enterovirus, HRV	19	0	108
GSE68310	Zhai	GPL10558	WB	USA	Adults with ARIs		Influenza, HRV, RSV, Enterovirus, Coronavirus	98	0	75
GSE82050	Tang	GPL21185	WB	Germany	Adults with influenza		Influenza	15	0	24

Table S2. Validation datasets of the transcriptomic signatures. Independent human datasets were used to test the diagnostic classifier. Information on the source of the data and the patient cohorts that were used to test the classifier are listed.

Accession	Author	Platform	Tissue	Location	Demographic	Bacteria	Viruses	# of healthy samples	# of bacterial samples	# of viral samples
E-MTAB-5195	Jong	GPL570	WB	Netherlands	Infants with RSV		RSV	4	0	39
GSE103842	Rodriguez-Fernandez	GPL10558	WB	USA	Young children hospitalized with bronchiolitis		RSV	12	0	62
GSE30119	Banchereau	GPL6947	WB	USA	Children with community acquired Staph infection	S. aureus		44	10	0
GSE34205	Ioannidis	GPL570	PBMC	USA	Children with ARIs		Influenza, RSV	22	0	79
GSE4607	Wong	GPL570	WB	USA	Septic children in the PICU	Multiple	Influenza	15	9	2
GSE6269 GPL96	Ramilo	GPL96	PBMC	USA	Children with bacterial or viral sepsis	S. aureus, S. pneumoniae	Influenza	6	12	3
GSE66099	Sweeney	GPL570	WB	USA	Septic children in the PICU	Multiple	Influenza, HMPV, Parainfluenza	47	35	5
GSE67059 GPL10558	Heinonen	GPL10558	WB	USA, Spain, Finland	Previously healthy children with asymptomatic or symptomatic HRV		HRV	16	0	20
GSE67059 GPL6947	Heinonen	GPL6947	WB	USA, Spain, Finland	Previously healthy children with asymptomatic or symptomatic HRV		HRV	21	0	80
GSE73072 (RSV DEE1)	Liu	GPL14604	WB	USA	Patients in the acute phase of a viral challenge study		RSV	20	0	9
GSE73072 (H3N2 DEE2)	Liu	GPL14605	WB	USA	Patients in the acute phase of a viral challenge study		Influenza	17	0	9
GSE73072 (H1N1 DEE3)	Liu	GPL14606	WB	USA	Patients in the acute phase of a viral challenge study		Influenza	22	0	9
GSE73072 (H1N1 DEE4)	Liu	GPL14607	WB	USA	Patients in the acute phase of a viral challenge study		Influenza	19	0	5
GSE73072 (H3N2 DEE5)	Liu	GPL14608	WB	USA	Patients in the acute phase of a viral challenge study		Influenza	21	0	8
GSE73072 (HRV UVA)	Liu	GPL14609	WB	USA	Patients in the acute phase of a viral challenge study		HRV	20	0	8
GSE73072 (HRV DUKE)	Liu	GPL14610	WB	USA	Patients in the acute phase of a viral challenge study		HRV	26	0	11
GSE77087	de Steenhuijsen Piters	GPL10558	WB	USA	Young children with mild and severe RSV disease		RSV	23	0	81

Table S3. Quenched fluorescent probe formulations of ABN panel. Substrate sequences were incorporated into quenched fluorescent probes. 5FAM = Fluorescein, CPQ2 = Quencher, PEG2 = polyethylene glycol. The other capital letters are single amino acid codes.

Substrate	Sequence
BV01-F	(5FAM)-GGAIEFDSGK(CPQ2)-(PEG2)-C
BV02-F	(5FAM)-GGHPGGPQGK(CPQ2)-(PEG2)-C
BV03-F	(5FAM)-GGGVFRMLSVGK(CPQ2)-(PEG2)-C
BV04-F	(5FAM)-GGGLFRSLSSGK(CPQ2)-(PEG2)-C
BV05-F	(5FAM)-GGGLLYGKGGK(CPQ2)-(PEG2)-C
BV06-F	(5FAM)-GGy-Tic-TNGK(CPQ2)-(PEG2)-C
BV07-F	(5FAM)-GGfPRSGGGK(CPQ2)-(PEG2)-C
BV08-F	(5FAM)-GGGSGRSANAKGK(CPQ2)-(PEG2)-C
BV09-F	(5FAM)-GGGIQQRSLGGGK(CPQ2)-(PEG2)-C
BV10-F	(5FAM)-GGIPSIQSRGLGK(CPQ2)-(PEG2)-C
BV11-F	(5FAM)-GGNLARALKQTIGK(CPQ2)-(PEG2)-C
BV12-F	(5FAM)-GGHMVQHLIQWHGK(CPQ2)-(PEG2)-C
BV13-F	(5FAM)-GGPRAAA-Homophe-TSPGK(CPQ2)-(PEG2)-C
BV14-F	(5FAM)-GGTGPPGYTGK(CPQ2)-(PEG2)-C
BV15-F	(5FAM)-GGTGLPVYQGK(CPQ2)-(PEG2)-C
BV16-F	(5FAM)-GG-Nle(O-Bzl)-Met(O)2-Oic-Abu-K(CPQ2)-(PEG2)-C
BV17-F	(5FAM)-GGAAFAGK(CPQ2)-(PEG2)-C
BV18-F	(5FAM)-GGGGGPGK(CPQ2)-(PEG2)-C
BV19-F	(5FAM)-GGPLGMRGGK(CPQ2)-(PEG2)-C
BV20-F	(5FAM)-GGP-(Cha)-G-Cys(Me)-HAGK(CPQ2)-(PEG2)-C

Table S4. Recombinant proteases and buffers used for *in vitro* screens. Specific buffers were used to create optimal cleavage conditions for each recombinant protease. Activation buffers were used for pre-incubation of the protease as needed.

Name	Acronym	Product info	Assay buffer	Activation buffer
Fibroblast Activation Protein	FAP	R&D (3715-SE)	50 mM Tris, 1 M NaCl, 1 mg/mL BSA, pH 7.5	
Neutrophil Elastase	NE	Enzo BML-SE284-0100	0.1 M Tris-HCl pH 8	
Legumain	LGMN	R&D (2199-CY)	50 mM MES, 250 mM NaCl, pH 5.0	50 mM Sodium Acetate, 100 mM NaCl, pH 4.0
Matrix metalloproteinase 9	MMP9	Enzo (BML-SE360)	50 mM Tris, 0.15 M NaCl, 10 mM CaCl2, 0.05% Brij-35 (w/v), pH 7.5	
Proteinase 3	PR3	Enzo (BML-SE498-0025)	100mM MOPS pH 7.5, 500mM NaCl, 10% DMSO, 100μM DTNB	
Matrix metalloproteinase 24	MMP24	Enzo (ALX-201-105-C010)	50mM TRIS-HCl, pH 7.57, 150mM NaCl, 5mM CaCl2, 0.025% Brij 35.	
Granzyme K	GZMK	Enzo (ALX-201-117-C010)	50 mM TRIS, pH 8.0, 0.15M NaCl, 0.01% Triton X-100, 0.3mM DTNB	
Granzyme A	GZMA	R&D (2905-SE)	50 mM Tris, pH 8.0	0.1 M Tris, pH 9.0
SARS-CoV-2 3CL Protease	3CLpro	R&D (E-720)	50 mM HEPES, 0.1 M NaCl, pH 8	
Cathepsin B	CTSB	R&D (953-CY-010)	25 mM MES, pH 5.0	25 mM MES, 5 mM DTT, pH 5.0
Napsin A	NAPSA	R&D (8489-NA-050)	50 mM Sodium Acetate, 100 mM NaCl, pH 4.0	
Granzyme H	GZMH	R&D (1377-SE-010)	50 mM Tris, 1 M NaCl, 1 mg/mL BSA, pH 7.5	
Cathepsin G	CTSG	Enzo (BML-SE283-0100)	160 mM Tris-HCl, 1.6 M NaCl, pH 7.7	
SARS-CoV-2 GST-Papain-like Protease	PLpro	R&D (E-611)	50 mM HEPES, 0.1 M NaCl, pH 8	
A Disintegrin and Metalloprotease-like Domain 9	ADAM9	R&D (939-AD-020)	25 mM Tris, 2.5 μM ZnCl2, 0.005% (w/v) Brij-35, pH 9.0	
Granzyme B	GZMB	R&D (2906-SE)	50 mM Tris, pH 7.5	50 mM MES, 50 mM NaCl, pH 5.5
Matriptase	ST14	R&D (3946-SEB-010)	50 mM Tris, 0.05% (w/v) Brij-35, pH 9.5	
Human Coagulation Factor II	F2	R&D (1473-SE-010)	50 mM Tris, 1 M NaCl, 1 mg/mL BSA, pH 7.5	
Cathepsin K	CTSK	Enzo (BML-SE553-0010)	25 mM MES, 5 mM DTT, pH 5.0	
Kallikrein 5	KLK5	R&D (1108-SE-010)	0.1 M NaH2PO4, pH 8.0	
Trypsin 3	PRSS3	R&D (3714-SE)	50 mM Tris, 0.15 M NaCl, 10 mM CaCl2, 0.05% Brij-35 (w/v), pH 7.5	
